# A glucose-starvation response governs endocytic trafficking and eisosomal retention of surface cargoes in budding yeast

**DOI:** 10.1242/jcs.257733

**Published:** 2021-01-25

**Authors:** Kamilla M. E. Laidlaw, Daniel D. Bisinski, Sviatlana Shashkova, Katherine M. Paine, Malaury A. Veillon, Mark C. Leake, Chris MacDonald

**Affiliations:** 1 York Biomedical Research Institute and Department of Biology, University of York, York, UK; 2 Department of Physics, University of York, York YO10 5DD, UK

**Keywords:** Eisosomes, Endosomal trafficking, Glucose starvation, Membrane trafficking

## Abstract

Eukaryotic cells adapt their metabolism to the extracellular environment. Downregulation of surface cargo proteins in response to nutrient stress reduces the burden of anabolic processes whilst elevating catabolic production in the lysosome. We show that glucose starvation in yeast triggers a transcriptional response that increases internalisation from the plasma membrane. Nuclear export of the Mig1 transcriptional repressor in response to glucose starvation increases levels of the Yap1801 and Yap1802 clathrin adaptors, which is sufficient to increase cargo internalisation. Beyond this, we show that glucose starvation results in Mig1-independent transcriptional upregulation of various eisosomal factors. These factors serve to sequester a portion of nutrient transporters at existing eisosomes, through the presence of Ygr130c and biochemical and biophysical changes in Pil1, allowing cells to persist throughout the starvation period and maximise nutrient uptake upon return to replete conditions. This provides a physiological benefit for cells to rapidly recover from glucose starvation. Collectively, this remodelling of the surface protein landscape during glucose starvation calibrates metabolism to available nutrients.

This article has an associated First Person interview with the first author of the paper.

## Abstract (precis)

**Summary:** Endocytosis of many cell surface proteins is triggered following glucose starvation, with recovery from starvation mediated by a small fraction of nutrient transporters preserved at the surface in eisosomes.

## INTRODUCTION

Cell surface membrane protein cargoes perform diverse roles, including initiating signal transduction pathways, uptaking nutrients and maintaining the ionic balance of the cell. Surface proteins are controlled by various complex and overlapping trafficking routes, many of which are conserved throughout evolution (Feyder et al., 2015). Endocytosis of surface proteins from the plasma membrane provides a layer of control, where cargoes can either be temporarily removed from the surface and recycled back or permanently removed via ubiquitin-mediated degradation in the lysosome (Laidlaw and MacDonald, 2018; MacDonald and Piper, 2016). In response to starvation, it is thought that many surface proteins are downregulated as a survival mechanism to reduce energy consumption via non-essential anabolic processes, whilst also increasing flux to the lytic lysosome for degradation and an increase in catabolic supply to the cell. How eukaryotic cells respond to changes in nutrients can be controlled at different levels, but mechanisms underlying many of these are not fully understood. Studies in yeast have revealed how large-scale surface protein degradation is mediated in response to restricted nutrients at late log phase (MacDonald et al., 2012a) or relatively severe starvation conditions lacking nitrogen or carbon (Lang et al., 2014; MacGurn et al., 2011; Müller et al., 2015). More specific nutrient starvation, such as depletion of vitamins or amino acids, trigger different trafficking responses to increase lysosomal degradation (Jones et al., 2012; MacDonald and Piper, 2017; MacDonald et al., 2015).

Clathrin-mediated endocytosis is the best characterised mechanism to package cargo into endocytic vesicles and is governed by dozens of different factors. Although we are yet to fully understand how these factors cooperate, many aspects about the function (Kaksonen and Roux, 2018) and sequential coordination (Cocucci et al., 2012; Kaksonen et al., 2003; Mund et al., 2018) of these endocytic proteins has been revealed. The process is initiated by cytosolic adaptors, like assembly polypeptide 2 (AP2), which coordinates cargo recruitment at specific lipid interaction sites, in addition to binding clathrin (Kelly et al., 2014). Other adaptors, such as epsin, clathrin assembly lymphoid myeloid leukemia protein (CALM, also known as PICALM) or its neuronal counterpart assembly protein 180 kDa (AP180, also known as SNAP91), also interact with surface lipids through defined domains early in the endocytic process (Ford et al., 2001; Itoh et al., 2001; Miller et al., 2015a). Recruitment of clathrin and assembly of these components alongside actin polymerisation and membrane-bending Bin-Amphiphysin-Rvs (BAR) domain proteins serve to generate the burgeoning vesicle, followed by enzyme driven scission (Kaksonen and Roux, 2018). Internalised proteins that retain a ubiquitylation signal are recognised by the endosomal sorting complexes required for transport (ESCRT) apparatus and delivered through the multivesicular body (MVB) pathway for degradation in the lysosome/yeast vacuole (Piper and Katzmann, 2007). Although endosomal organisation and recycling mechanisms in yeast are less clear (Day et al., 2018; Ma and Burd, 2019), proteins that are not destined for degradation can recycle back to the surface via different mechanisms involving either ubiquitylation or deubiquitylation (MacDonald and Piper, 2017; MacDonald et al., 2017; Xu et al., 2017).

Surface cargoes are organised spatially within the PM, such as nutrient transporters in yeast that diffuse into plasma membrane invaginations termed eisosomes (Bianchi et al., 2018; Grossmann et al., 2008; Spira et al., 2012). Eisosomes have recently been shown to regulate both lipids and proteins at the surface in response to stress (Babst, 2019). Plasma membrane tension is sensed by the eisosomal osmotic stress sensors Slm1 and Slm2, which subsequently activate TORC2 to alter lipid metabolism (Riggi et al., 2018). As to surface protein regulation, many nutrient transporters have been shown to localise to eisosomes, in particular during nutrient stress conditions when eisosomes maintain higher levels of these cargoes (Appadurai et al., 2019; Gournas et al., 2018; Grossmann et al., 2008; Moharir et al., 2018; Spira et al., 2012). Addition of transporter substrate results in a shift of transporters from eisosomes to other membrane compartments, where they can function to uptake nutrients before downregulation (Babst, 2019). It is not fully understood how eisosomes restrict access to endocytosis, but this affords cargo protection and preservation during mass downregulation.

Yeast has been a useful model to study physiological changes induced in response to changes in carbon source (Broach, 2012). Yeast preferentially uses glucose as a carbon source for fermentative growth, as with some rapidly growing mammalian cells (Diaz-Ruiz et al., 2011), but has also developed strategies to use various alternative carbon sources. During growth in replete glucose, genes from alternative carbon utilisation pathways, for example galactose (*GAL*), maltose (*MAL*) and sucrose (*SUC*) genes, are actively repressed (Gancedo, 1998). The glucose-sensitive transcriptional repressor Mig1, which binds consensus sequences in the promoter regions of these example genes, is responsible for this transcriptional repression (Griggs and Johnston, 1991; Hu et al., 1995; Nehlin and Ronne, 1990; Vallier and Carlson, 1994). Additionally, predicted Mig1 consensus binding sequences have been identified in promoters of other functionally diverse genes (Wollman et al., 2017). Gene expression profiles in mutant cells lacking *MIG1* and/or the related repressor *MIG2* also span various functional classes beyond sugar metabolism (Westholm et al., 2008). When glucose-starved cells are returned to glucose, alternative carbon transporters are endocytosed and rely on ubiquitylation for degradation, which is provided by spatially distinct, cognate arrestin-related trafficking adaptors (ARTs) at the surface and endolysosomal system (Becuwe and Léon, 2014; Becuwe et al., 2012; Hovsepian et al., 2018). Recent work has also shown how high-affinity glucose transporters, which are not required during glucose starvation, are specifically endocytosed and degraded via Rsp5 and the Csr2 ART adaptor, which is repressed by Mig1/Mig2 in glucose-rich conditions (Hovsepian et al., 2017). It is less clear whether such transcriptional and posttranslational regulatory mechanisms described for these sugar transporters during acute glucose starvation also control other surface membrane proteins.

In this study, we show that glucose privation alleviates Mig1-mediated repression of yeast clathrin AP180 adaptors, which increases endocytosis to more efficiently downregulate surface cargoes. We also find that eisosomal factors are transcriptionally upregulated during glucose starvation in a Mig1-independent manner. Specific eisosomes increase in size to sequester a small portion of nutrient transporters, which requires the presence of Ygr130c. We propose a model where this reserve pool of surface-localised nutrient transporters provide a physiological benefit upon a return to nutrient-replete conditions, which outweighs the advantages of degrading an entire population of existing nutrient transporters.

## RESULTS

### Glucose depletion triggers cargo downregulation

**Fig. 1.**
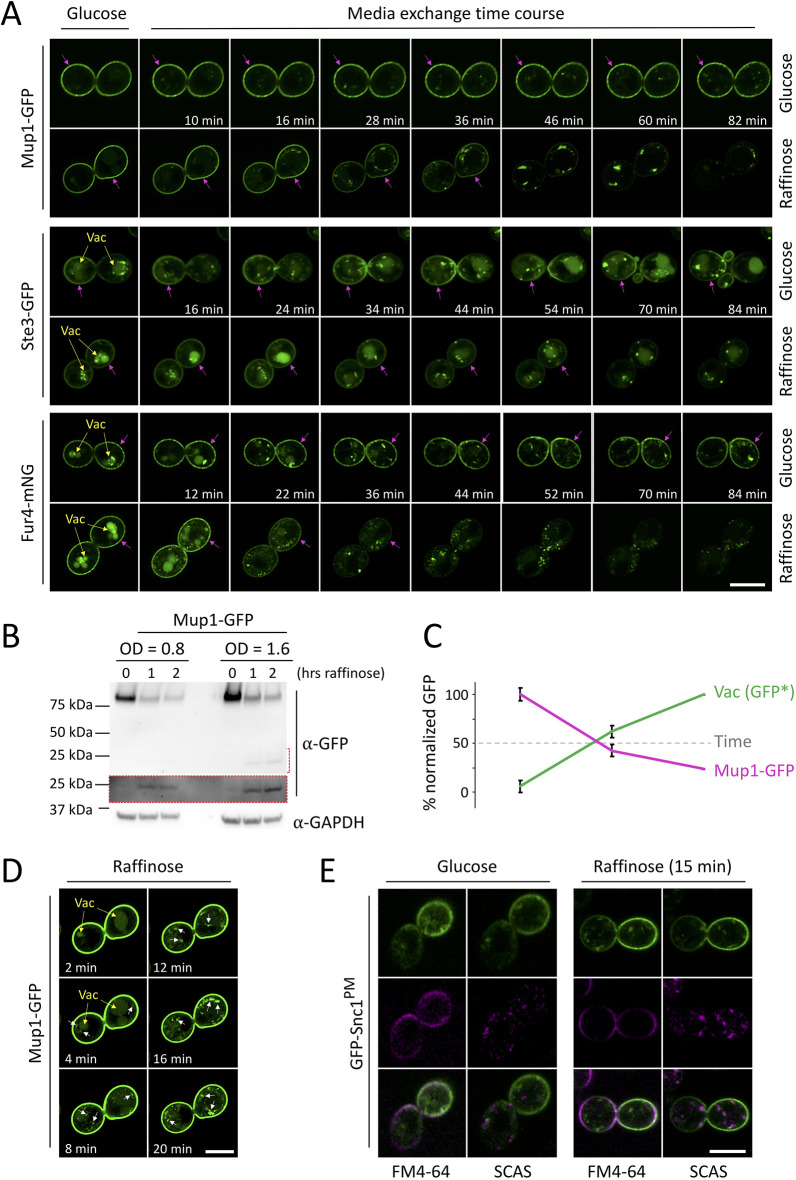
**Glucose starvation results in downregulation of surface proteins.** (A) Wild-type cells expressing the indicated labelled cargoes were grown to mid-log phase, processed for time-lapse microscopy and then imaged every 2 min in either glucose or raffinose media for the indicated timecourse. Vacuoles are indicated with yellow arrows, and magenta arrows denote significant plasma membrane signal. (B) Mup1–GFP levels in glucose- (0 hrs raffinose) and raffinose-treated cells first grown to the indicated densities (OD_600_) were assessed by immunoblotting. Increased exposure (red box) of the region indicated by a red dashed bracket shows levels of vacuolar processed GFP. Loading was assessed using anti-GAPDH antibodies. (C) Graph depicts percentage full-length Mup1–GFP versus vacuolar processed GFP [Vac (GFP*)] following raffinose addition over time, as described for B. Mean±s.d. from *n*=3. (D) Increased exposure of early time points from the experiment in A, to show internalised Mup1–GFP (white arrows). (E) Wild-type cells expressing GFP–Snc1^PM^ at log phase were incubated with glucose or raffinose for 30 min prior to flushing with FM4-64-containing media for 5 min, then exchanging with media containing 2.4 µM SCAS prior to imaging. FM4-64 labelling is shown in magenta. Scale bars: 5 µm.

We developed a microfluidic system to rapidly replace glucose with raffinose medium whilst monitoring the localisation of the methionine permease Mup1, a useful reporter for endocytosis (MacDonald et al., 2012a). We provided the trisaccharide raffinose, which undergoes slow enzymatic conversion outside the cell first (de la Fuente and Sols, 1962), to minimise changes in osmotic potential whilst inhibiting glucose-dependent signal transduction pathways. Extended incubations in glucose medium had little effect on the surface localisation of Mup1 (Fig. 1A; Movie 1). However, glucose starvation triggered efficient Mup1–GFP endocytosis, with most signal internalised after ∼45 min, following similar trafficking kinetics to substrate induced endocytosis (Fig. S1A). Photobleaching during time-lapse microscopy did not affect endosomal GFP-tagged cargo but did reduce observable vacuolar signal in the confocal plane (Fig. S1B). Therefore, Mup1 endocytosis and delivery to the vacuole for degradation was also assessed biochemically, with immunoblots of vacuolar processed GFP showing a large increase upon glucose starvation (Fig. 1B,C). Raffinose exchange also induced downregulation of functionally and structurally distinct surface proteins, including the G-protein-coupled receptor Ste3 tagged with GFP, the uracil permease Fur4 tagged with mNeonGreen (mNG), and the arginine permease Can1 tagged with GFP (Fig. 1A; Fig. S1C, Movie 2). However, the ATP-binding cassette (ABC) transporter Yor1, which is downregulated in response to NAD^+^ starvation (MacDonald et al., 2015), showed no endocytosis, even at extended raffinose incubations (Fig. S1D). Furthermore, although relatively long exposures to lactate medium triggers vacuolar delivery of high-affinity hexose transporters tagged with GFP, we observed no sorting of GFP–Hxt6 or GFP–Hxt7 during raffinose treatment (Fig. S1E). This suggests that glucose depletion simulates rapid downregulation of many, but not all, surface proteins, and demonstrates that raffinose provides a glucose-starvation condition without carbon source withdrawal. Raffinose treatment rapidly triggers relocalisation of Mup1–GFP to bright endosomal puncta at very short time points (e.g. 4 min), prior to sorting to the vacuolar lumen (Fig. 1D). Additionally, endocytic uptake of FM4-64 at short time points was assessed in cells expressing a mutant of GFP–Snc1^PM^ that is defective in endocytosis and therefore a surface marker (Lewis et al., 2000). Although raffinose-treated cells displayed lower levels of FM4-64 dye binding (Fig. S1F), possibly due to changes in the lipid composition of the plasma membrane, we found efficient endocytosis in glucose-depleted conditions, with significant FM4-64 internalised following 4 min uptake (Fig. 1E). We conclude glucose starvation triggers large-scale endocytosis and downregulation of surface proteins.

### Mig1 regulates endocytic genes during starvation

**Fig. 2.**
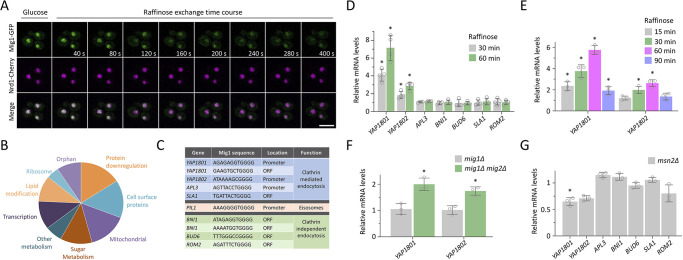
**Mig1 and glucose regulate expression of clathrin adaptor genes.** (A) Time-lapse microscopy of wild-type cells co-expressing Mig1–mGFP and Nrd1–mCherry grown in glucose medium prior to exchange with raffinose medium for the indicated times. (B) Pie chart showing *in silico* categorisation of Mig1 binding targets in the yeast genome. (C) Table of putative Mig1 target genes previously shown to function in endocytosis. (D–G) Total RNA was prepared and transcript levels measured by RT-qPCR of the indicated genes, using *ACT1* and *HEM2* genes as controls, from the indicated cells grown to mid-log phase in glucose medium. Comparing raffinose exchange for 30 and 60 min (D) or 15, 30, 60 and 90 min (E) with glucose controls, or comparing *mig1*Δ and *mig1*Δ *mig2*Δ cells (F) and *msn2*Δ cells (G) with wild-type cells. Jitter plots show mean±s.d. *n*=3–4 experiments, each with three technical replicates. *P<0.05 (unpaired Holm–Sidak *t*-test). Scale bar: 5 µm.

We investigated a potential role for Mig1, which represses many genes in glucose-replete conditions (Nehlin and Ronne, 1990; Nehlin et al., 1991), in cargo endocytosis following glucose starvation. Mig1 repression in the nucleus is alleviated by its translocation to the cytoplasm upon medium exchange for alternative carbon sources (De Vit et al., 1997), including raffinose (Fig. 2A; Movie 3). A consensus Mig1 binding sequence, containing motif (G/C)(C/T)GGGG, from validated promoters (Lundin et al., 1994) has been used to screen promoter regions (−500 bp upstream of start codon) to identify 106 Mig1-target genes (Wollman et al., 2017). Potential Mig1 targets were clustered based on function, revealing many factors associated with protein downregulation (Fig. 2B), including roles in ubiquitylation, autophagy and vacuolar degradation (Table S1). This list also included several endocytosis factors as potential Mig1 targets (Fig. 2C), including genes encoding clathrin adaptors Yap1801, Yap1802, and Apl3 (Goode et al., 2015). We also noticed that other endocytic genes contained consensus Mig1 binding sequences in their open-reading frame (ORF) sequences, so included these in downstream transcriptomic analyses. Conditions for qPCR were optimised for each potential Mig1 target gene (Fig. S2A–C) to reveal that the yeast AP180 clathrin adaptors were upregulated in raffinose medium. Most prominently, *YAP1801* exhibited a robust ∼4-fold increase in expression levels 30 min after raffinose addition, which increased to ∼6 fold at 60 min (Fig. 2D). *YAP1802* levels also increased significantly, with a sustained ∼2-fold increase following starvation incubations. Transcriptional upregulation of yeast AP180 adaptors was relatively acute, with transcript levels returning close to basal levels 90 min after raffinose (Fig. 2E), a period sufficiently long to largely downregulate many cargoes (Fig. 1). We found that *mig1*Δ *mig2*Δ cells showed elevated levels of *YAP1801* and *YAP1802* transcript levels when compared to levels in wild-type cells (Fig. 2F). There was no appreciable change in clathrin adaptor levels in *mig1*Δ cells, with it being necessary to also delete *MIG2*, which encodes a protein that has many of the same regulatory elements as Mig1 (Westholm et al., 2008) but is not translocated from the nucleus in responsive to glucose depletion (Fig. S2D). We also found decreased *YAP1801* and *YAP1802* transcript levels in *msn2*Δ null cells (Fig. 2G), which lack the Msn2 regulator that is known to activate many of the genes that Mig1 represses (Lin et al., 2015). This indirect evidence further supports a role of Mig1 in *YAP1801* and *YAP1802* expression, because expression of all other genes unaffected by raffinose treatment was unchanged in *msn2*Δ cells.

### Glucose-sensitive AP180 adaptors trigger endocytosis

**Fig. 3.**
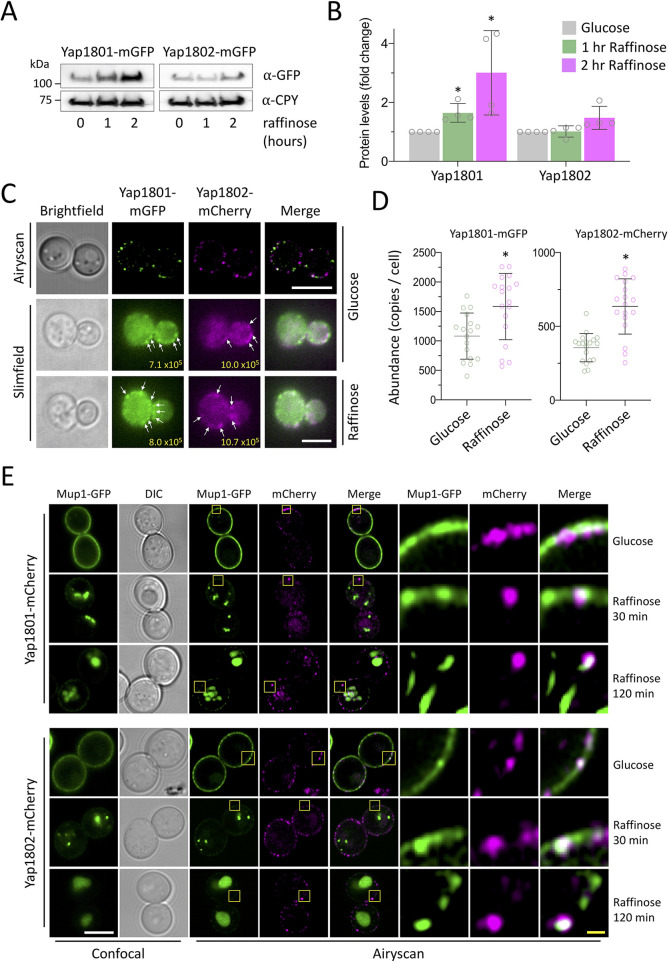
**Yap1801** **and Yap1802 proteins are upregulated during glucose starvation.** (A) Immunoblot analysis of endogenously tagged Yap1801–mGFP and Yap1802–mGFP in wild-type cells grown in glucose and raffinose media. Anti-CPY antibody was used as a loading control. (B) Histogram showing average Yap1801–mGFP and Yap1802–mGFP intensity, normalized to intensity in glucose medium. Mean±s.d. of *n*=4. (C) Wild-type cells expressing Yap1801–mGFP and Yap1802–mCherry at mid-log phase were imaged by Airyscan confocal and Slimfield microscopy. For Slimfield, bright foci are indicated with white arrows, and the integrated density for diving cells in each frame is shown in yellow. These data represent the first of 200 frames, acquired every 5 ms, with representative full datasets shown in Movie 3. (D) Numbers of Yap1801 and Yap1802 molecules per cell (>20 cells) were estimated using autofluorescence- and background-corrected integrated density values obtained using ImageJ software. Mean±s.d. is shown. (E) Airyscan confocal colocalisation of Mup1–GFP with Yap1801–mCherry (upper panels) and Yap1802–mCherry (lower panels) in glucose and raffinose media. Yellow boxes denote zoomed-in regions shown in the panels to the right. *P<0.05 (unpaired Holm–Sidak *t*-test). Scale bars: 5 µm (white), 1 µm (yellow).

The AP180 proteins are conserved early acting endocytic factors that bind phosphatidylinositol 4,5-bisphosphate [PI(4,5)P_2_] at the plasma membrane, via their AP180 N-terminal homology (ANTH) domain, to recruit clathrin to endocytic sites (Ford et al., 2001; Wendland and Emr, 1998). We endogenously tagged the yeast AP180 adaptors with mGFP and confirmed that raffinose exchange resulted in a significant increase in Yap1801–mGFP, and a modest increase in Yap1802–mGFP levels (Fig. 3A,B). Yeast AP180 proteins showed punctate surface localisation, with both distinct and overlapping localisations (Fig. 3C; Fig. S3A). These confocal experiments indicated an increase in fluorescence intensity following acute raffinose treatment (Fig. S3B–D), but we sought a more accurate method to study endocytosis in living yeast cells. For this, we employed single-molecule Slimfield microscopy (Fig. S3E, Movie 4), using a narrow field of laser excitation (Wollman and Leake, 2015), which enabled precise spatial resolution and millisecond-scale time sampling (Badrinarayanan et al., 2012; Reyes-Lamothe et al., 2010). This imaging system utilises spatially delimited illumination confined to the vicinity of a single cell and allows for detection of fluorescently labelled proteins directly in living yeast cells (Shashkova et al., 2020; Wollman et al., 2017). Based on the background and autofluorescence-corrected intensity of each cell, and the fluorescence intensity of a single mGFP and mCherry molecule, we were able to estimate the protein copy numbers within the cell. Levels of Yap1801 and Yap1802 were estimated to be ∼1000 and ∼400 molecules per cell, respectively, similar to previously reported estimates (Ho et al., 2018), with a significant increase in abundance for both Yap1801 and Yap1802 following glucose starvation (Fig. 3D). Endogenously mCherry-labelled versions of Yap1801 and Yap1802 were then used to monitor their localisation in relation to cargo in cells co-expressing Mup1–GFP. In replete conditions, Mup1 levels were dispersed across the plasma membrane, overlapping with spots of both Yap1801 and Yap1802 (Fig. 3E). Most Mup1–GFP signal was internalised following raffinose treatment (Fig. 1), but Airyscan confocal microscopy revealed that ∼2.0±1.3% (mean±s.d.) Mup1–GFP localised to punctate surface structures after raffinose exchange (Fig. S3F), including AP180-colocalised protein, presumably destined for internalisation, and cargo sequestered in eisosomes (discussed below).

**Fig. 4.**
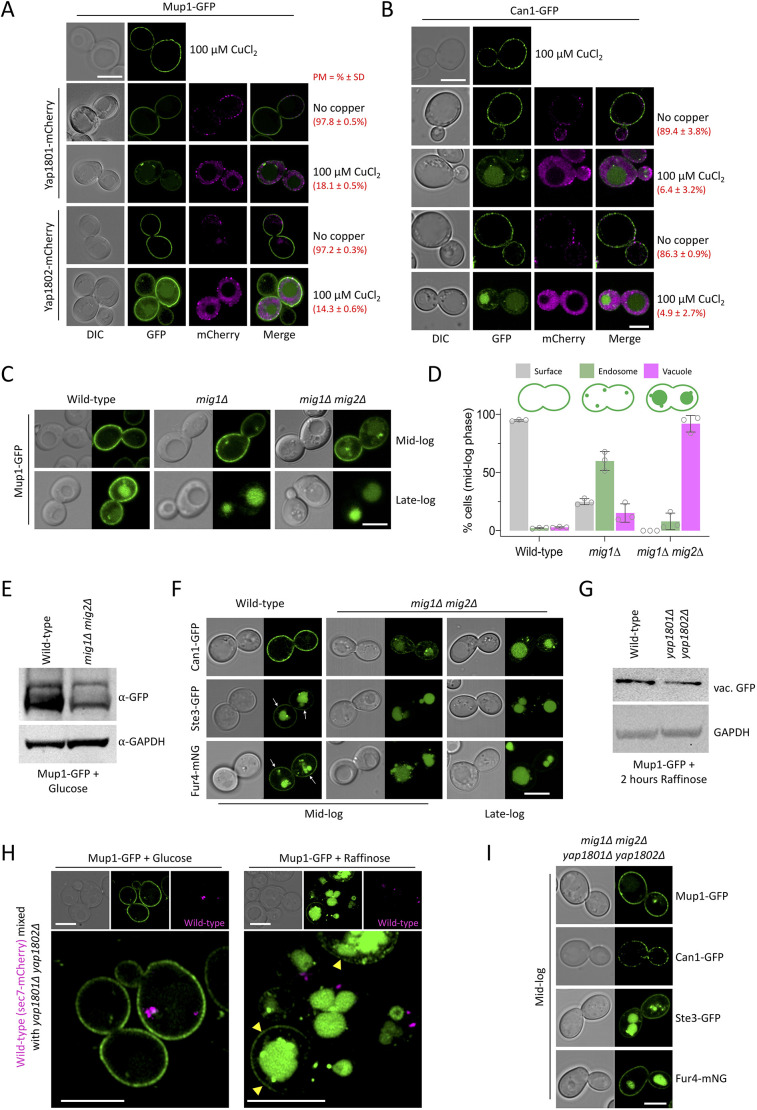
**Increased Yap1801/Yap1802 increases rates of cargo endocytosis.** (A,B) Airyscan confocal microscopy imaging of wild-type cells co-expressing Mup1–GFP (A) or Can1–GFP (B) and Yap1801–mCherry or Yap1802–mCherry, expressed under control of the copper-inducible *CUP1* promoter. The mean±s.d. percentages of cells exhibiting exclusive plasma membrane localisation for each condition (>75 cells per conditions) are shown in red text. (C) Localisation of Mup1–GFP in wild-type, *mig1*Δ, and *mig1*Δ *mig2*Δ cells at mid- and late-log phase. (D) Quantification of the experiment shown in C, with 35–120 mid-log phase cells quantified from each condition. Mean±s.d. *n*=3. (E) Immunoblot analysis of Mup1–GFP expressed in wild-type and *mig1*Δ *mig2*Δ cells. GAPDH was used as a loading control. (F) Wild-type and *mig1*Δ *mig2*Δ cells expressing Can1–GFP, Ste3–GFP and Fur4–mNG were imaged at mid- and late-log phase. Surface-localised signal is indicated with white arrows. (G) Immunoblot analysis of vacuolar GFP from lysates generated from wild-type and *yap1801*Δ *yap1802*Δ cells expressing Mup1-GFP. GAPDH was used as a loading control. (H) Mup1–GFP was expressed in wild-type cells labelled with Sec7–mCherry and in unlabelled *yap1801*Δ *yap1802*Δ cells, mixed in a 1:1 ratio and co-cultured for 2 h, followed by 90 additional minutes in glucose (left) or raffinose (right) media prior to fluorescence microscopy. Surface-localised signal is indicated with yellow arrowheads. (I) Quadruple-null *mig1*Δ *mig2*Δ *yap1801*Δ *yap1802*Δ cells expressing Mup1–GFP, Can1–GFP, Ste3–GFP or Fur4–mNG were imaged at mid-log phase. Scale bars: 5 µm.

Our model predicts that elevated levels of Yap1801 and Yap1802 induced upon glucose starvation are sufficient to upregulate endocytosis. To test this, mCherry tagged AP180 adaptors were expressed from a plasmid under the control of the copper inducible *CUP1* promoter. With no addition of copper, low levels of Yap1801–mCherry or Yap1802–mCherry, similar to endogenously tagged versions, had no effect on the surface localisation of co-expressed Mup1–GFP or Can1–GFP (Fig. 4A,B). However, overexpression of mCherry tagged Yap1801 and Yap1802 was sufficient to trigger endocytosis. The yeast AP180 adaptors have been reported to have a range of localisation patterns (Burston et al., 2009; Carroll et al., 2012; Newpher et al., 2005; Yamamoto et al., 2017), but we did not find any particular localisations (Fig. S4A) that correlated with specific endocytic events, only that elevated levels were sufficient to increase endocytosis. Furthermore, Mup1–GFP endocytosis was induced slightly in *mig1*Δ cells, and substantially more in *mig1*Δ *mig2*Δ cells (Fig. 4C–E), sorting of which is further accelerated when cells are grown to late log phase (MacDonald et al., 2012a, 2015). Similarly, endocytosis of Can1–GFP, Ste3–GFP and Fur4–mNG was elevated in *mig1*Δ *mig2*Δ cells (Fig. 4F), presumably through the increased levels of Yap1801 and Yap1802. As expected, endocytosis in *mig1*Δ *mig2*Δ cells was phenocopied in cells lacking the upstream Reg1 component, but not in cells lacking the low-glucose sensor Snf3 (Fig. S4B). Efficiency of endocytosis upon raffinose exchange was attenuated in *yap1801*Δ *yap1802*Δ cells, shown by Mup1–GFP sorting to the vacuole (Fig. 4G) and internalisation from the surface (Fig. 4H) being reduced. Endocytosis of Mup1–GFP induced by growth to late log phase was also reduced (Fig. S4C). Furthermore, as endocytosis of Mup1, Can1, Ste3 and Fur4 induced by deletion of *MIG1* and *MIG2* was ablated by the further deletion of *YAP1801* and *YAP1802* (Fig. 4I), we conclude that Mig1 and Mig2 repress transcription of *YAP1801* and *YAP1802* in glucose-replete conditions, which is lifted in response to glucose starvation to trigger clathrin-mediated endocytosis of a broad range of cargoes.

### Eisosomal reorganisation sequesters cargo during glucose starvation

**Fig. 5.**
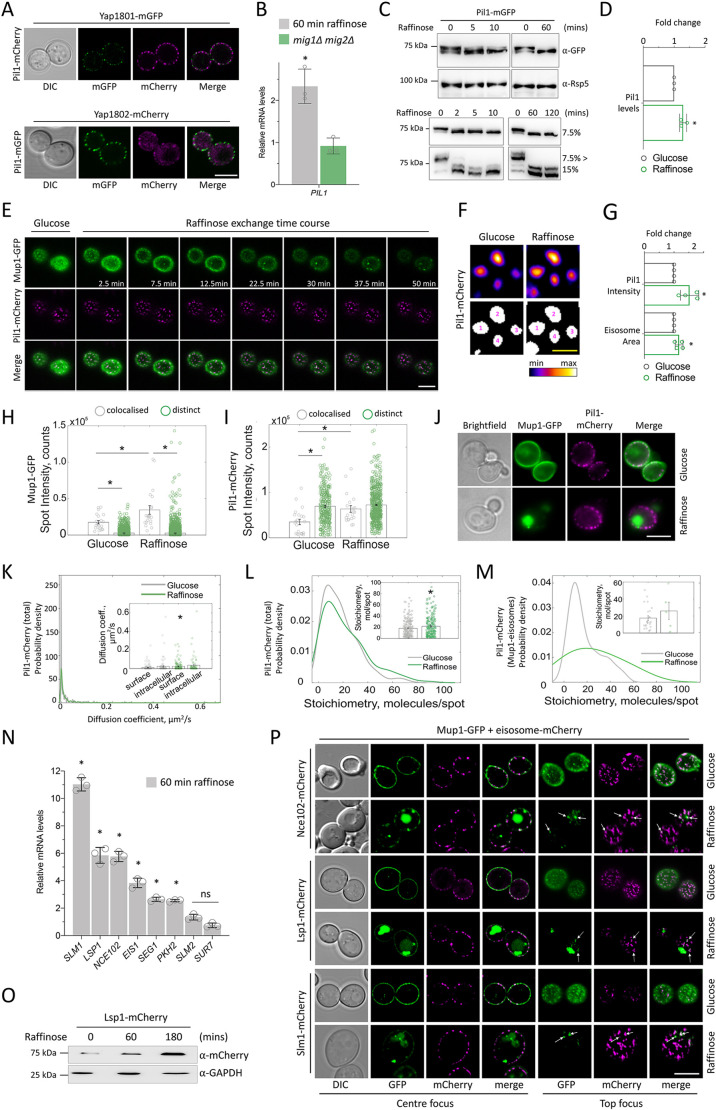
**Eisosomes sequester surface proteins in glucose-starvation conditions.** (A) Fluorescence microscopy of cells expressing Yap1801–mGFP and Pil1–mCherry, or Yap1802–mCherry and Pil1–GFP. (B) RT-qPCR of *PIL1* was performed using RNA extracted from wild-type cells grown in glucose with or without 1 h in raffinose medium (grey) or from wild-type and *mig1*Δ *mig2*Δ cells grown in glucose (green). Mean±s.d. from *n*=3 biological replicates (each with *n*=3 technical replicates). (C) Pil1–GFP levels in wild-type cells over a raffinose timecourse assessed by immunoblotting following resolution on standard (upper panels and lower panels, 7.5% acrylamide) and step-gradient (lower panels, 7.5%>15% acrylamide) resolving gels. Anti-Rsp5 was used as a loading control. (D) Quantification of total Pil1–GFP levels from immunoblots (*n*=3) using cells grown in glucose or raffinose for 1 h. Mean±s.d. (E) Average intensity projections of top-focussed Airyscan confocal images shown for a raffinose exchange timecourse of wild-type cells co-expressing Pil1–mCherry and Mup1–GFP. (F) Pil1–mCherry intensity from time-lapse microscopy in glucose and raffinose compared with a gradient-based density look-up table (upper panels) and segmented using identical Li thresholding parameters (17/65,535) to create numbered eisosome regions of interest (ROIs; lower panels). (G) ROIs generated in F were used to calculate average Pil1–mCherry intensities and eisosome areas. Mean±s.d. (H,I) Jitter plots of fluorescence intensities of Mup1–GFP (H) and Pil1–mCherry (I) spots measured for both colocalised (grey) and distinct (green) foci from cells grown to mid-log phase and either prepared directly for confocal microscopy (glucose) or first grown in raffinose media for 30 min. Mean±s.e.m. (J) Examples of the first frame of Slimfield acquisitions from cells co-expressing Mup1–GFP and Pil1–mCherry in glucose (upper panel) and raffinose solid media conditions (lower panel). (K–M) Kernel density plots of Pil1–mCherry foci diffusion coefficient distribution (K), whole-cell Pil1–mCherry stoichiometry distribution (L) and Pil1–mCherry stoichiometry distribution from only foci that colocalise with Mup1–GFP (M) are shown from cells incubated in glucose (grey) or raffinose (green) media. Insets: jitter plots of diffusion coefficients and stoichiometries. Mean±s.d. (N) RT-qPCR of the indicated genes was performed from RNA extracted from wild-type cells grown in glucose followed by 60 min raffinose exchange. Mean±s.d from *n*=3 biological replicates (each averaged from three technical replicates). (O) Levels of integrated Lsp1–mCherry was assessed using anti-mCherry immunoblotting of lysates generated from growth in raffinose medium for the indicated periods, with GAPDH blot as a loading control. (P) Airyscan confocal microscopy of cells expressing the indicated mCherry-tagged eisosome proteins and co-expressing Mup1–GFP from a plasmid, after growth in glucose or 45 min in raffinose. Arrows indicate colocalisation of sequestered cargo with eisosome marker. *P<0.05; ns, not significant (Student's *t*-test). Scale bars: 5 µm (white); 1 µm (yellow).

Spatial mapping and functional analyses of surface cargoes have recently shown that lipid domains, termed eisosomes, harbour surface proteins and antagonise their endocytosis (Bianchi et al., 2018; Busto et al., 2018; Gournas et al., 2018; Moharir et al., 2018). Pil1 is required for proper formation of eisosomes (Walther et al., 2006) and *PIL1* was implicated as a potential Mig1 target, we therefore examined its role in harbouring cargo in eisosomes. As expected, fluorescently tagged Pil1 failed to colocalise with sites of endocytosis marked by Yap1801 or Yap1802 (Fig. 5A). Upregulation of eisosomes that contravene cargo endocytosis may seem contradictory in conditions that also upregulate endocytosis; however, cargo protection in eisosomes during starvation and osmotic shock has recently been documented (Appadurai et al., 2019; Gournas et al., 2018). We found that *PIL1* was regulated at the transcriptional level in response to raffinose treatment, but we observed no difference in expression in *mig1*Δ *mig2*Δ cells (Fig. 5B; Fig. S5A), suggesting Pil1 protein levels are significantly increased during glucose starvation (Fig. 5C,D) through a Mig1/2-independent mechanism. To test whether this transcriptional upregulation of eisosomes impacted cargo endocytosis we performed time-lapse microscopy of cells co-expressing Mup1–GFP cargo and Pil1–mCherry, to mark eisosomes. We optimised 4D confocal conditions to best observe eisosomes at the surface (Fig. S5B) before initiating glucose starvation by flushing raffinose medium using microfluidics. Mup1–GFP colocalised with eisosomes in glucose-replete medium (Fig. 5E), with the majority of Mup1–GFP being distributed at the plasma membrane. As shown, the bulk of Mup1–GFP is endocytosed following glucose starvation (Fig. 1); however, we noticed concentration of Mup1–GFP within some, but not all, eisosomal structures (Fig. 5E). This is in contrast to when the Mup1 substrate methionine was added, which triggers flux from eisosomes to distinct plasma membrane regions that allow internalisation (Busto et al., 2018), implying that this response is related to starvation of the cell and not a mechanism associated with typical transporter regulation. As Mup1–GFP was observed to diffuse out of eisosomes during initial starvation, eisosome targeting may be a stochastic process; it is curious that only certain eisosomes appear to accumulate Mup1–GFP, with no signal observed for other Pil1-marked compartments, suggesting a precise regulatory mechanism. In support of this idea, we captured dramatic reconfigurations of Pil1–mCherry in eisosomes retaining Mup1–GFP (Movie 5). We noted that Pil1-marked eisosomes increased in size and intensity over the course of time-lapse microscopy following raffinose exchange (Fig. 5F,G), so we performed a series of steady-state experiments to fully quantify this phenotype, which confirmed that the intensity of Mup1–GFP foci at eisosomes increased significantly following glucose starvation, with no change in intensity of Mup1–GFP molecules that were in other, non-eisosomal regions of the cell (Fig. 5H; Fig. S5C). Similarly, in raffinose grown cells, Pil1–mCherry intensity was unchanged in regions that lacked Mup1–GFP, but increased in eisosomes harbouring cargo (Fig. 5I).

Although we observed an increase in Pil1 levels, at whole-cell level and at eisosomes, during raffinose exchange, we observed no change in eisosome number (Fig. S5D), implying the increased levels of Pil1 localise to and regulate existing eisosomes to better sequester cargo, as shown by confocal microscopy (Fig. 5F,G). Interestingly, we found that Pil1 was rapidly dephosphorylated upon raffinose treatment (Fig. 5C); our optimised SDS-PAGE (MacDonald et al., 2020) revealed a marked shift from slower to faster migrating dephosphorylated bands that have been previously correlated with increased Pil1 assembly at eisosomes (Walther et al., 2007). Mup1–GFP- and Pil1–mCherry-expressing cells, in both glucose and raffinose media, were used for Slimfield imaging (Fig. 5J; Movie 6), which allowed acquisition of hundreds of images per field of view at 5 ms exposure times enabling a lateral localisation precision of ∼40 nm until no fluorescence signal could be detected (Miller et al., 2015b). We then applied a bespoke MATLAB code for single-particle tracking to identify all fluorescent spots within one image and link them to those on the next image, so that trajectories of foci movements could be built for further diffusion and stoichiometry (number of molecules per bright spot) analysis (Leake et al., 2006; Shashkova and Leake, 2017). As fluorescent proteins photobleach in a stepwise manner, we applied previously optimised protocols for stoichiometry estimations by comparing intensity of each identified fluorescent spot with a single fluorophore intensity. Single-molecule analysis of plasma membrane-localised proteins showed that, although there were no physiological changes in Mup1–GFP (Fig. S5E,F), there was an increase in the diffusion coefficient (Fig. 5K) and molecular stoichiometry (Fig. 5L) of Pil1–mCherry. It may be that increased Pil1 intensity represents an increase in eisosome size, or just more Pil1 molecules per eisosome. Either way, the molecular response to starvation serves to better sequester cargo. Also, although most Mup1–GFP molecules were no longer found within the surface region following starvation, the few spots retained showed that the stoichiometry of Pil1 in these regions increased (Fig. 5M). Collectively, this helps rationalise how a relatively modest increase in *PIL1* transcription and protein levels, alongside its rapid posttranslational modification, allow a subset of eisosomes to sequester Mup1–GFP during glucose starvation. Beside this, we also documented more dramatic raffinose-induced increases in expression of other eisosomal genes. Similar to *PIL1*, the levels of *PKH2* and *SEG1* were increased by ∼2.5-fold, but others showed a larger increase, with mRNA levels of *NCE102* and *LSP1* increasing ∼6-fold and *SLM1* levels ∼11-fold after 60 min raffinose (Fig. 5N). We found that levels of Lsp1 increased significantly during raffinose exchange (Fig. 5O), and the phenotype of Mup1–GFP localising to specific eisosomes was found with mCherry-labelled eisosome proteins Nce102, Lsp1 and Slm1 (Fig. 5P). We therefore believe that eisosomes are relatively stable and that the transcriptional upregulation of factors triggers modulation of existing eisosomes to concentrate cargo during acute glucose starvation.

### Ygr130c is required for efficient cargo retention at eisosomes

**Fig. 6.**
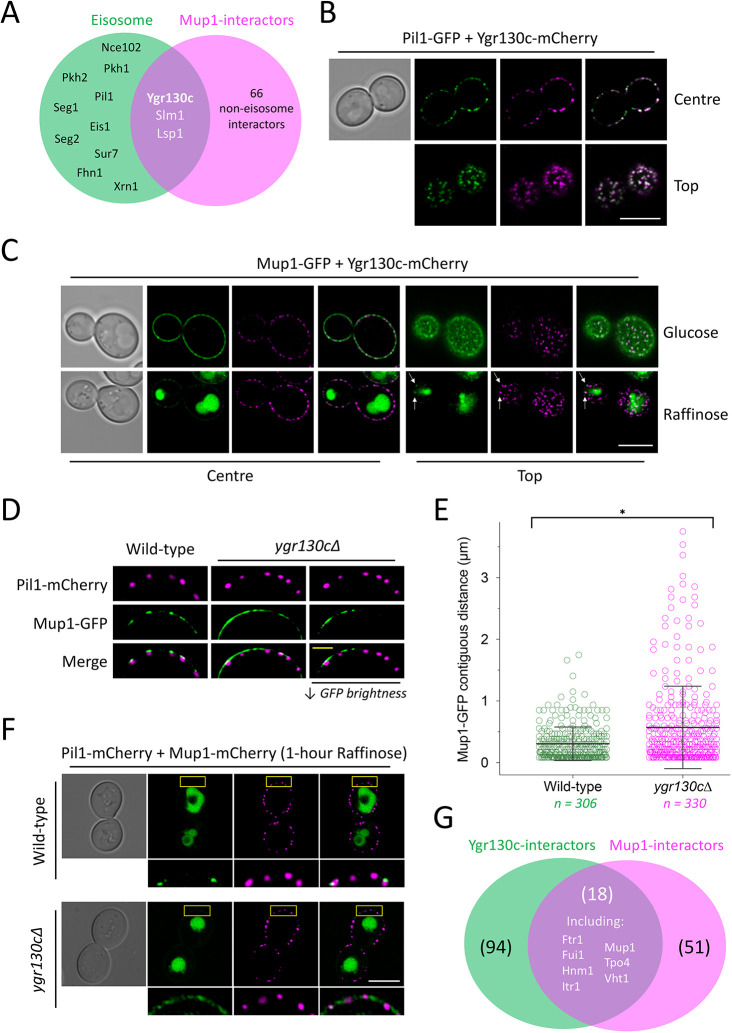
**Ygr130c is required for efficient eisosomal cargo retention.** (A) Venn diagram showing overlap between known eisosome proteins (green) and Mup1 physical interactors (magenta). (B) Cells co-expressing Pil1–GFP (green) and Ygr130c–mCherry (magenta) were imaged at centre and top focus planes using confocal microscopy. Merge images are shown on the right. (C) Cells expressing Ygr130c–mCherry (magenta) were transformed with a Mup1–GFP (green) plasmid and imaged in glucose or raffinose media by Airyscan confocal microscopy. Merge images are shown on the right for each condition. Arrows indicate spots of colocalisation. (D) Colocalisation of Mup1–GFP- and Pil1–mCherry-marked eisosomes in wild-type and *ygr130c*Δ mutants was recorded by Airyscan confocal imaging. A panel of reduced GFP brightness is included to better show GFP distribution (right). (E) Contiguous plasma membrane GFP signal (in µm) was measured in wild-type (green) and *ygr130c*Δ cells (magenta). Mean±s.d. (F) Wild-type and *ygr130c*Δ cells co-expressing Pil1–mCherry (magenta) and Mup1–GFP (green) following 1 h of raffinose exchange were compared using Airyscan microscopy. Boxes indicate regions shown in enlarged images below. (G) Venn diagram between physical interactors of Ygr130c (green) and Mup1 (magenta); 18 proteins are present in both sets. *P<0.05 (Student's *t*-test). Scale bars: 5 μm (white), 1 μm (yellow).

As Mup1–GFP appears to target only a subset of eisosomes during glucose starvation, we performed bioinformatics analyses to identify possible eisosome factors that physically interact with the cargo Mup1 (Tables S2,S3) and might serve as eisosome anchors. Of ∼70 known physical Mup1 interactions, three were eisosome factors: Lsp1, Slm1 and Ygr130c (Fig. 6A). The functional roles of Lsp1 (Walther et al., 2006; Ziółkowska et al., 2011) and Slm1 (Audhya et al., 2004; Fadri et al., 2005) have been characterised, and we found that these proteins colocalise with all Pil1-marked eisosomes (Fig. S5G) and behave as Pil1 during starvation-induced cargo retention (Fig. 5P). Therefore, we focussed on the potential role of Ygr130c. Although identified as an eisosomal-resident protein from a visual screen (Grossmann et al., 2008), and shown to interact with the PI(4,5)P_2_ phosphatase Inp51, along with other eisosome factors (Fröhlich et al., 2014), the function of Ygr130c remains unknown; although it has been implicated in regulation of chronological lifespan through an unknown mechanism (Yucel and Ulgen, 2011). We tagged Ygr130c with mCherry and found that it colocalises with Pil1–GFP eisosomes (Fig. 6B) and Mup1–GFP in both glucose and raffinose conditions (Fig. 6C). To test the hypothesis that Ygr130c could act as a physical eisosomal anchor for Mup1, we expressed Mup1–GFP in mutant *ygr130c*Δ cells and found that its distribution across the plasma membrane was more dispersed than in wild-type cells, where Mup1–GFP was found to colocalise with distinct Pil1–mCherry-marked eisosomes (Fig. 6D). We developed a method to calculate the size of Mup1–GFP signals at the plasma membrane (Fig. S6), which showed a statistically significant increase in contiguous length of Mup1–GFP signals at the surface of *ygr130c*Δ cells, indicating reduced residency in eisosomes (Fig. 6E). This was more evident following glucose starvation, where after 1 h raffinose exchange, surface-retained Mup1 was primarily colocalised with Pil1–mCherry puncta (Fig. 6F). In contrast, Mup1–GFP signal was more diffuse at the surface of glucose-starved *ygr130c*Δ cells, and the most concentrated signal was observed in regions distinct from Pil1–mCherry. We propose the role of Ygr130c is to increase residency of Mup1 diffusing into eisosomes, which is particularly important to retain a reserve pool of Mup1 during glucose starvation. Beyond Mup1, other surface nutrient transporter proteins that physically interact with Ygr130c have been identified, including uridine (Fui1), inositol (Itr1) and polyamine (Tpo4) transporters (Table S2). Furthermore, Ygr130c interactors include nutrient transporters for iron (Ftr1), choline (Hnm1), and biotin (Vht1), and these cargoes have been previously shown to colocalise to some degree (coefficients of 0.09±0.01, 0.08±0.01, and 0.08±0.01, respectively) with the eisosome marker Sur7 (Spira et al., 2012). This retention mechanism therefore may extend to additional nutrient transporters and might explain why a relatively high number of known Mup1 interactors are also known to physically bind Ygr130c (Fig. 6G).

### Eisosomes provide a recovery growth benefit following starvation

**Fig. 7.**
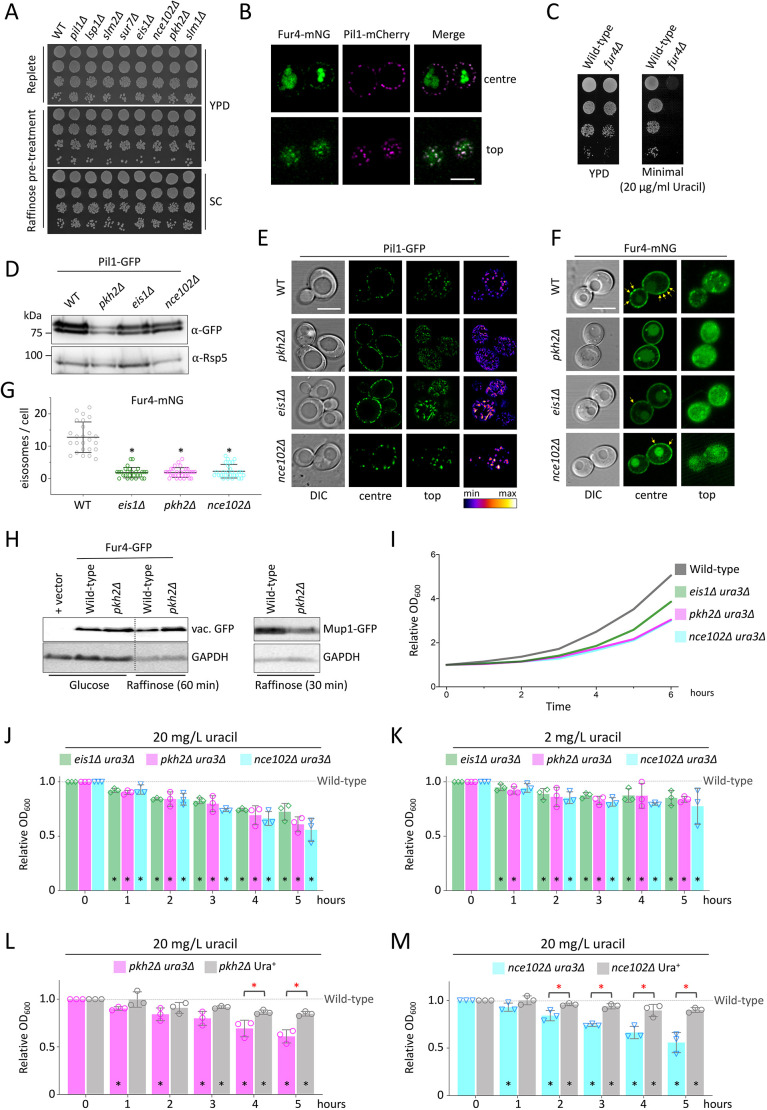
**Eisosomes are required for efficient recovery following glucose starvation.** (A) Mid-log cultures of the indicated yeast strains were incubated in glucose (replete) or raffinose media for 2 h, before plating on YPD or SC plates for two days at 30°C. (B) Colocalisation of Pil1–mCherry and Fur4–mNG, by Airyscan confocal microscopy focussed at the centre and top, in cells grown to mid-log phase before imaging in minimal medium. (C) Growth of wild-type and *fur4*Δ cells grown in rich medium prior to serial dilution and growth on YPD and SC (minimal) solid media for 2 days at 30°C. (D) Levels of Pil1–GFP expressed from a plasmid in the indicated strains were assessed by immunoblot of lysates generated from mid-log phase cells using anti-GFP antibodies, with anti-Rsp5 antibodies used as a loading control. (E) Pil1–GFP localisation of transformants from D grown to log phase was recorded by confocal microscopy at centre- and top-focussed planes. Images on the right show cells coloured using a lookup table of GFP intensity. (F) The indicated strains were transformed with a plasmid expressing Fur4–mNG from its endogenous promoter and imaged by confocal microscopy. Yellow arrows on centre-focussed images indicate pronounced eisosomal concentrations. (G) The numbers of eisosomally localised Fur4–mNG puncta per cell were quantified from the indicated yeast strains. Mean±s.d. (H) Levels of Fur4–GFP sorted to the vacuole (left) and full-length Mup1–GFP (right) were assessed from indicated strains and conditions by anti-GFP immunoblotting, using GAPDH as a loading control. (I) Indicated strains were grown to mid-log phase in SC complete medium before harvesting and growth in raffinose medium for two hours, before average recovery growth was assessed by OD_600_ measurements from cells incubated in SC medium. (J–M) Average recovery from assay described in I was quantified relative to the wild-type control and plotted over time for the indicated mutants at either 20 mg/l uracil (J,L,M) or 2 mg/l uracil (K). Mean±s.d. from three biological replicates. Black asterisks indicate Student's *t*-test comparisons with wild-type samples, red asterisks indicate comparisons between strains; *P<0.05. Scale bars: 5 µm.

Not all surface cargoes localise to eisosome compartments, for example the receptor Ste3–GFP, and a version retained at the plasma membrane (Ste3–GFP-DUb) showed no colocalisation with Pil1–mCherry (Fig. S7A). We propose that eisosomes increase in size or structure specifically to better harbour nutrient transporters, to persist through glucose-starvation periods and provide a physiological benefit upon a return to glucose-replete conditions. We reasoned that cells deficient in this eisosome function would exhibit growth deficiencies by virtue of their inability to maximise nutrient uptake. However, growth on rich and synthetic defined media, and estimated doubling times, showed no obvious difference between wild-type and null cells lacking various eisosome components (Fig. 7A; Fig. S7B). Despite this, we did observe that some mutants, including *pil1*Δ, *lsp1*Δ and *nce102*Δ, failed to reach the optical density of wild-type cells upon growth to saturation (Fig. S7C), implying their nutrient uptake capacity might be compromised. The eisosomal-localised permease Fur4 (Fig. 7B) is required for the uptake of uracil (Jund and Lacroute, 1970) and growth of *ura3*Δ auxotroph strains in minimal medium with low uracil concentrations (Fig. 7C). We reasoned that Fur4-mediated uracil scavenging following glucose starvation would provide a recovery-based assay with a dynamic range to test the role of eisosomes. For this, we used *pkh2*Δ cells, which have reduced levels of unmodified and phosphorylated Pil1 (Fig. 7D), and redistributed Pil1 localisation into reticular structures at the plasma membrane (Fig. 7E) reminiscent of structures observed in *pkh2*Δ cells carrying a temperature-sensitive allele of *pkh1* (Walther et al., 2007). We also used *eis1*Δ and *nce102*Δ mutants, which are defective in eisosome assembly and cargo retention (Aguilar et al., 2010; Fröhlich et al., 2009; Loibl et al., 2010) (Fig. 7E; Fig. S7D). These eisosome mutants had a decreased amount of Fur4–mNG localised to eisosomes (Fig. 7F,G) and showed higher rates of cargo endocytosis in both glucose and raffinose conditions (Fig. 7H; Fig. S7E), allowing us to test the hypothesis that cargo storage in eisosomes aids recovery from nutrient stress. Log-phase cells were exchanged with raffinose medium for 2 h before return to replete medium of varying concentrations of uracil, and growth was recorded over time (Fig. 7I). The recovery rate of growth for wild-type cells was significantly better than that of all three null mutants with defective eisosomes, across all concentrations of uracil tested (Fig. 7J,K; Fig. S7F,G) or when cells were returned to medium with only 10% amino acid levels (Fig. S7H). This benefit can be attributed in part to Fur4-mediated uracil uptake, as uracil-prototroph eisosome mutants recovered better from glucose starvation compared with their auxotrophic counterparts (Fig. 7L,M; Fig. S7I). However, there was still a statistically significant reduction in recovery of eisosome mutants prototrophic for uracil compared with recovery of the Ura^+^ wild type, so we conclude that, whilst Fur4 is an important cargo sequestered in eisosomes to facilitate recovery following starvation, it is not the only nutrient transporter required for this response.

## DISCUSSION

**Fig. 8.**
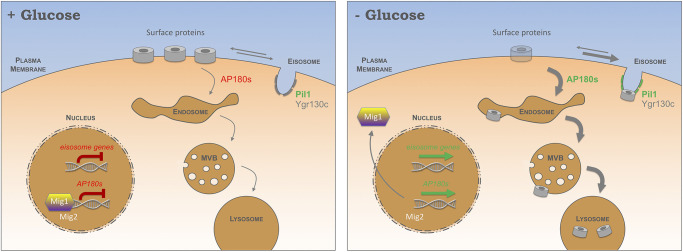
**Model for modes of surface protein regulation in response to glucose.** Schematic diagram shows the regulation of cell surface membrane protein cargoes in response to glucose starvation. When cells have access to glucose for maximal growth, internalisation occurs at a basal level through Mig1 (and Mig2) repression of endocytic adaptor genes *YAP1801/YAP1802*. Whilst resident at the surface, nutrient transporters diffuse in and out of eisosomes. The expression of many eisosome genes are repressed in glucose conditions (left). Following glucose starvation, Mig1 translocates from the nucleus, resulting in increased expression of *YAP1801/YAP1802*, which triggers increased cargo endocytosis and ubiquitin-mediated trafficking through the multivesicular body (MVB) pathway to the lysosome for degradation (right). Although most nutrient transporters are degraded following glucose starvation, a small reserve pool is sequestered in eisosomes, which are transcriptionally activated during starvation. Efficient cargo retention relies on Ygr130c, and eisosomes that specifically retain cargo during glucose starvation exhibit biochemical and biophysical changes in Pil1. This reserve pool of eisosomally retained nutrient transporters can be deployed upon a return to glucose-replete conditions for efficient recovery following the period of glucose starvation.

Yeast cells provided with appropriate carbon and ammonium can synthesise all amino acids for protein translation; however, amino acid biosynthesis and transporter-mediated uptake is coordinated with multiple metabolic programmes, including sugar utilisation (Ljungdahl and Daignan-Fornier, 2012). We find the replacement of glucose with raffinose, a suboptimal alternative carbon source, induces endocytosis and degradation of various surface proteins unrelated to sugar metabolism (e.g. amino acid transporters). We propose that a glucose-sensitive transcriptional response mediates surface cargo downregulation (Fig. 8). In glucose-replete conditions, endocytosis is maintained at a basal level, in part through the transcriptional repression via Mig1 (and Mig2) of the clathrin adaptor genes *YAP1801* and *YAP1802*. When glucose is exchanged for raffinose, Mig1 translocates from the nucleus, resulting in elevated levels of cellular Yap1801 and Yap1802, which coincides with accelerated endocytosis (Fig. 4). Although AP180 adaptor null mutants exhibited only a modest effect in cargo endocytosis upon glucose depletion, their deletion in a *mig1*Δ *mig2*Δ background supressed endocytosis. Our interpretation of this data is that AP180 adaptors are not essential for starvation-induced endocytosis, but their increased expression (by either glucose starvation, *MIG1 MIG2* deletion, or plasmid overexpression) triggers a cascade of events, likely involving much of the canonical endocytic machinery, that work collaboratively to package and internalise an array of surface proteins. In addition to lipid-binding domains, Yap1801 and Yap1802 associate with cortical clathrin (Newpher et al., 2005) and Pan1, which is required for endocytosis (Maldonado-Báez et al., 2008) and in turn activates Arp2/3-mediated actin assembly (Duncan et al., 2001; Wendland and Emr, 1998; Wendland et al., 1999). As the disordered domains of AP180 have been shown to amplify membrane curvature (Zeno et al., 2018), higher levels of these proteins are reasonable candidates for initiating a relatively broad downregulation of cargo following glucose replacement. This scenario best explains our observations that various cargoes are downregulated following increased levels of AP180 adaptors (in raffinose medium, in *mig1*Δ *mig2*Δ cells and during plasmid-borne overexpression), as deletion of these adaptors, as with many other yeast endocytic factors, does not lead to a strong defect in endocytosis (Huang et al., 1999; Wendland and Emr, 1998) or only cargo-specific defects (Burston et al., 2009).

We note that the increases in AP180 adaptor transcript levels in raffinose is relatively modest (up to 6-fold), which presumably can coordinate meaningful upregulation of clathrin-mediated endocytosis. This is in contrast to overexpression of AP180 (Ford et al., 2001) and its homologue CALM (Tebar et al., 1999) from plasmid-borne CMV promoter constructs in mammalian cells, which inhibits endocytosis; we presume this level of overexpression serves to sequester clathrin in a manner disruptive to multiple processes, including internalisation. The finding that FM4-64 internalises efficiently in raffinose (Fig. 1E) but not in sugar-free medium (Lang et al., 2014) may be explained by (1) the lack of carbon source triggering shutdown of internalisation to protect ATP maintenance – a process not necessary in the presence of raffinose, which can be hydrolysed by invertase – or (2) changes in plasma membrane tension and/or osmotic pressure in medium lacking sugar triggering TORC2-mediated changes in lipid composition (Appadurai et al., 2019; Riggi et al., 2018) that affect FM4-64 binding. The finding that elevated endocytosis downregulates surface proteins conceptually aligns with a starvation model wherein existing resources are degraded to supply essential processes whilst non-essential processes are turned off. Increasing endosomal residency of surface proteins will enhance cargo ubiquitylation at late endosomes, which is sufficient to trigger ESCRT recruitment, luminal vesicle formation and subsequent cargo sorting through the MVB pathway (MacDonald et al., 2012b). Endosomal cargo ubiquitylation is largely mediated by the E3 ubiquitin ligase Rsp5 in complex with cargo-specific adaptors, some of which are also regulated in response to glucose via adenosine monophosphate-activated protein kinase (AMPK) (O'Donnell and Schmidt, 2019). As certain Rsp5 adaptors, such as Rod1, Sna3 and Hua1, have been proposed to function later in the endocytic pathway, downstream of internalisation (Hovsepian et al., 2018; MacDonald et al., 2012a, 2020) these adaptors may be more important for downregulation when glucose is replaced with an alternative carbon source.

One initially contrary finding is that eisosomes, which antagonise endocytosis (Gournas et al., 2018; Grossmann et al., 2008; Moharir et al., 2018; Spira et al., 2012), contain many factors that are transcriptionally upregulated in raffinose (Fig. 5B,N), suggesting that surface cargoes are controlled within subdomains of the plasma membrane. This finding can be rationalised by recent models that propose cargo protection in response to nutritional stress retains surface proteins for later use (Gournas et al., 2018; Moharir et al., 2018). Similarly, although our data shows that surface proteins are largely endocytosed and degraded in response to raffinose, there is a small population, for example Mup1–GFP (Fig. 5E), that are retained and concentrated within eisosomes in these conditions. Increased Pil1 intensity following raffinose exchange, in addition to our 4D confocal microscopy capturing structural rearrangements of Pil1–mCherry at eisosomes that most significantly trap Mup1–GFP (Movie 5), suggests significant conformational changes occur in response to raffinose, with eisosomes increasing in size and possibly deepening to better sequester cargo. This also explains the initially confounding result that the diffusion coefficients from our single-molecule analysis of Pil1–mCherry increased upon raffinose treatment (Fig. 5K). Although the increase in Pil1 protein levels was a modest ∼29% increase, we note Pil1 is abundant, with different techniques providing estimates of 1.1×10^5^–2.7×10^5^ molecules per cell (Ghaemmaghami et al., 2003; Lahtvee et al., 2017; Tkach et al., 2012), suggesting that subtle changes could supply the cell with significant Pil1 at these cargo-capturing eisosomes. These subtle changes in Pil1 levels and molecular stoichiometry are accompanied by cellular Pil1 molecules undergoing rapid dephosphorylation during this period of starvation (Fig. 5C), which in combination may modify eisosomes to better retain cargo following glucose starvation.

Although single-cell stoichiometry estimates for Pil1 showed an increase upon raffinose treatment, the single-eisosome analysis revealed that the most significant changes occurred at eisosomes that retain cargo. Notably, nitrogen starvation and the broad nutritional stress of incubating cells for 12 h after they reach stationary phase, following complete exhaustion of nutrients, results in an increase in eisosomal numbers (Gournas et al., 2018). Unlike this severe starvation condition, our study reveals that glucose starvation specifically triggers an acute response (<2 h) that modulates Pil1 at the transcriptional, biochemical and biophysical level to effect cargo retention during this initial starvation period. This suggests cargo retention is tuneable to nutrient availability and specific starvation conditions may trigger specific retention responses, for example a preference for specific cargoes. As removal of carbon source results in a decrease in Nce102 at eisosomes (Appadurai et al., 2019), we predict that raffinose treatment, which increases transcript levels of *NCE102* around 6-fold, triggers a distinct response to specifically upregulate eisosomes to retain nutrient transporters during the metabolic challenge of suboptimal sugars. A central finding of our study is that the sequestration of amino acid transporters provides a physiological benefit to cells during changes in sugar availability, by protecting a reservoir of nutrient transporters during glucose-limited conditions. Upon a return to more favourable nutritional conditions, wild-type cells grow better than mutants that fail to properly sequester cargo in eisosomes (Fig. 7). In particular, Fur4-mediated uracil uptake is important for recovery, which might be explained by its apparent high affinity (Fig. 7C) and the fact that strains used in this study, and in many lab settings, are uracil auxotrophs (Brachmann et al., 1998; Pronk, 2002). Our results imply that the cell senses acute starvation broadly or the transporters that diffuse within eisosomes are retained indiscriminately of their potential to uptake specific nutrients. We also assign a function for the Mup1-binding eisosomal protein Ygr130c for the first time (Fig. 6), as an eisosomal anchor for Mup1 and possibly other nutrient transporters thought to diffuse within eisosomes (Babst, 2019). Although *ygr130c*Δ cells do not exhibit an obvious increase in nutrient transporter endocytosis, their diffusion within eisosomes is greatly reduced. It would be intriguing if future work assessed potential structural changes of eisosomes in these mutants. Our bioinformatics analysis suggests that many other nutrient transporters may also use Ygr130c to efficiently sequester in eisosomes (Fig. 6G) and may function with Fur4 to achieve full recovery from starvation (Fig. 7).

The finding that *YAP1801* and *YAP1802* are regulated in response to glucose via Mig1 and Mig2 shows that membrane trafficking factors are modulated to adapt to changes in sugar availability. We did note that the levels of *YAP1801* and *YAP1802* were elevated more when cells were acutely shifted to raffinose than when wild-type cells were compared with *mig1*Δ *mig2*Δ mutants. This might be explained by additional transcriptional regulators contributing to the glucose starvation response, or by *mig1*Δ *mig2*Δ mutants having acquired additional mutations to compensate for the systemic loss of Mig1 and Mig2. As gene deletions are often associated with secondary mutations, many of which affect stress responses (Teng et al., 2013), this latter idea is reasonable for *mig1*Δ cells, which are defective in various processes (Santangelo, 2006). Increases in yeast *AP180* gene expression following raffinose were 2.8±1.0-fold (mean±s.d.) higher than increases observed in *mig1Δ* *mig2Δ* cells. If this was also true of *PIL1* expression levels, which only showed a modest increase even in raffinose, any increase in *mig1Δ mig2Δ* cells might be so small as to be insignificant. (Fig. 5B). Alternatively, as glucose starvation induces expression of many eisosomal factors (Fig. 5N), such as *NCE102*, *PKH2*, *SLM1*, *LSP1*, *EIS1* and *SEG1*, that were not predicted to be transcriptionally repressed by Mig1/Mig2, *PIL1* may also be a Mig1-independent gene target regulated in response to glucose levels. This transcriptional response, alongside the induction of the AP180 clathrin adaptors described above, allows for acute metabolic control in response to fluctuating nutrient conditions. Many elements of this control involve proteins conserved throughout evolution, so we propose that similar regulation of these pathways and membrane subdomains in other eukaryotes could maintain metabolism in response to changes in extracellular nutrients.

## MATERIALS AND METHODS

### Reagents

Yeast strains and plasmids are listed in Table S4 and Table S5, respectively. The *mig1*Δ *mig2*Δ and *mig1*Δ *mig2*Δ *mig3*Δ yeast strains were a generous gift from Hans Ronne (Swedish University of Agricultural Sciences). Fluorescently labelled Vps4 strains were a gift from David Teis (Innsbruck Medical University). Polyclonal antibodies raised against GFP (Urbanowski and Piper, 1999) and Rsp5 (Stamenova et al., 2004), and monoclonal glyceraldehyde 3-phosphate dehydrogenase (GAPDH; clone 6C5; Ambion) and carboxypeptidase Y (CPY; clone 10A5-B5; Molecular Probes) antibodies, were used for immunoblot analysis. These antibodies were routinely used at 1:1000 dilution.

### Cell culture

Yeast were grown in rich yeast extract peptone dextrose (YPD) medium (2% glucose, 2% peptone, 1% yeast extract) or synthetic complete (SC) minimal medium (2% glucose, yeast nitrogen base supplemented with appropriate amino acid and base dropout mixtures; Formedium, Norfolk, UK) for maintaining of plasmids. Cells were routinely grown overnight to early/mid-log phase (OD_600_≤1.0) prior to experimental procedures, unless otherwise stated. To minimise nutritional challenges prior to starvation experiments, very low density cultures (OD_600_=0.1) were adhered to concavalin A-treated coverslips for time-lapse microscopy. For glucose-starvation experiments, rich and minimal media were supplemented with 2% raffinose instead of glucose, but were otherwise identical. Geneticin (Formedium), used at a concentration of 250 μg/ml in rich media, and methotrexate (Alfa Aesar), used at a working concentration of 20 mM, were prepared in SC minimal medium as described previously (MacDonald and Piper, 2015). Expression of proteins from the *CUP1* promoter was achieved by addition of 50–100 µM CuCl_2_ to the medium for at least 1 h prior to downstream analysis.

### Confocal microscopy

Cells were grown to mid-log phase and prepared for fluorescence microscopy experiments by concentration in minimal medium before imaging, or from a slide or glass-bottom dish using laser scanning confocal microscopes (LSM710 or LSM880 equipped with an Airyscan module; Zeiss) with a 63× Differential Interference Contrast (DIC) objective with a 1.4 numerical aperture. Argon laser excitation – 488 nm with emission filter set to 495–550 nm (for GFP, mGFP and mNeonGreen) and 561 nm with 570–620 nm emission filter (for mCherry and FM4-64) – was used.

### Microfluidics and time-lapse microscopy

Cells were grown to early log phase (approximately OD_600_=0.2) overnight and then adhered to 35 mm concavalin A-coated glass-bottom dishes (Ibidi GmbH, Germany) prior to live-cell imaging at room temperature. Plates were prepared by adding 1 mg/ml concavalin A in water to a coverslip for 5 min prior to several wash steps; plates were routinely stored at 4°C. Sterile media exchanges were performed using syringes through tubing fused to the lid of 35 mm dishes. Live cells were labelled with 0.8 µM FM4-64 using microfluidics for 2–5 min before flushing with medium containing 2.4 µM 4-sulfonato calix[8]arene sodium salt (SCAS; Biotium, Hayward, CA) for 30 s at room temperature to quench extracellular dye before further imaging.

### Image analysis

Micrographs were processed using Zeiss Zen and Fiji software. For time-lapse movies, bleach correction was carried out using a histogram-matching method (https://imagej.net/Bleach_Correction). For images of the top section of a yeast cell, five slices with 0.18 μm spacing were combined using the average intensity of the maximum intensity *z*-projection. Movies were made using time-lapse images of varying frame time points, as indicated in the movies. Numbers of eisosomes were identified using tracking software written in MATLAB and reported previously (Miller et al., 2015a; Wollman et al., 2017; Wollman and Leake, 2015).

### Slimfield microscopy and fluorescent foci analysis

Slimfield excitation was implemented via co-aligned 488 nm and 561 nm wavelength 50 mW lasers (OBIS, Coherent) de-expanded to direct a beam onto the sample full width at half maximum of ∼30 µm. For visualisation of both fluorophores, we employed rapid Alternating Laser Excitation (ALEX) with 5 ms exposure time per image frame for each laser (Syeda et al., 2019). Fluorescence emission was captured by a 1.49 NA oil immersion objective lens (Nikon), and subsequently split into separate green and red detection channels using a dual-pass green/red dichroic mirror centred at long-pass wavelength 560 nm combined with 25 nm bandwidth emission filters (Chroma) centred on 525 nm and 594 nm wavelengths. Each channel was imaged separately at 5 ms exposure time by an EMCCD camera (Prime 95B scientific CMOS; Teledyne Photometrics) using 50 nm/pixel magnification. The focal plane was set to mid-cell height using the bright-field appearance of cells. As photobleaching of mGFP and mCherry proceeded during Slimfield excitation, distinct fluorescent foci could be observed of width 250–300 nm, consistent with the diffraction-limited point-spread function of our microscope system, which were tracked and characterised in terms of their stoichiometry and apparent microscopic diffusion coefficient. Distinct fluorescent foci that were detected within the microscope's depth of field could be tracked for up to several hundred ms, to a super-resolution lateral precision σ=40 nm using a bespoke single-particle tracking algorithm. The molecular stoichiometry for each track was determined by dividing the summed pixel intensity values associated with the initial unbleached brightness of each foci by the brightness corresponding to that calculated for a single fluorescent protein molecule (either mGFP for 488 nm wavelength excitation or mCherry for 561 nm wavelength excitation) measured using a stepwise photobleaching technique described previously (Shashkova et al., 2018; Wollman et al., 2017). The apparent microscopic diffusion coefficient, D, was determined for each track by calculating the initial gradient of the relationship between the mean square displacement with respect to tracking time interval using the first five time interval values while constraining the linear fit to pass through 4σ^2^ on the vertical axis corresponding to a time interval value of zero*.* Cell body and membrane boundaries were detected based on the mCherry fluorescence images, considering the membrane width as 7 nm.

### Gene expression analysis

Yeast cultures (10 ml) were grown overnight to mid-log phase in YPD or exchanged with YPR (YPD containing 2% raffinose in place of glucose) for the indicated times prior to spheroplasting in yeast lysis buffer (1 M sorbitol, 100 mM EDTA, 0.1% β-mercaptoethanol) with 5 μl zymolase (5 U/μl; Zymo Research) added for 2 min at room temperature. For reverse transcription-qPCR (RT-qPCR), extraction of total RNA was performed using an RNeasy kit (QIAGEN) protocol with an additional DNaseI treatment, followed by a second DNaseI digestion with the TURBO DNA-free kit (Invitrogen). Extracted mRNA was then used for cDNA synthesis with the SuperScript IV reverse transcriptase (Invitrogen). 50 ng/μl random hexamers and 10 mM dNTPs were added to 5 μg RNA. Samples were incubated at 65°C for 5 min before addition of 100 mM DTT, ribonuclease inhibitor and the Superscript IV reverse transcriptase to initiate the reaction (10 min at 23°C; 10 min at 55°C; 10 min at 80°C), and then was used immediately for the qPCR reaction. For qPCR optimisation, two sets of primers targeting amplicons 70–170 bp in length were designed for each gene of interest, and the best performing were chosen for use in quantitation (Tables S6,S7). Single-product amplification was confirmed through PCR using genomic DNA, and near-100% amplification efficiencies were confirmed through qPCR on a standard curve of known input quantity, all performed in duplicate reactions (examples shown in Fig. S2). All qPCR reactions were performed on 20 ng cDNA, or relevant negative controls, in 20 µl reactions containing 350 nM forward and reverse primers and 10 µl Fast SYBR Green mastermix (Thermo Fisher Scientific). Reactions were carried out using the QuantStudio 3 system (Thermo Fisher Scientific) under the following conditions: 40 cycles of 95°C for 1 s and 60°C for 20 s before a continuous ramp from 60°C to 95°C at a rate of 0.1°C/s for melt-curve analysis. Gene expression levels were quantified using the comparative Ct (ΔΔCt) method, relative to the expression of the housekeeping gene (*ACT1* or *HEM2*) and were normalised to the control sample (i.e. wild type when testing mutant strains; glucose when assessing effects of raffinose timecourse). To avoid bias caused by averaging data that had been transformed through the equation 2^−Ct^ to give fold changes in gene expression, all statistics were performed on Ct values.

### Immunoblotting

Equivalent amounts of cells were harvested from early and late log phase (OD_600_=0.5 and 2.0, respectively) yeast cultures. Lysates were generated by alkali treatment with 0.2 M NaOH for 3 min prior to resuspension in lysis buffer (8 M urea, 10% glycerol, 50 mM Tris-HCl pH 6.8, 5% SDS, 0.1% Bromophenol Blue and 10% β-mercaptoethanol). Proteins were resolved using SDS-PAGE prior to transfer to a nitrocellulose membrane using the iBlot 2 dry transfer system (Thermo Fisher Scientific). Membrane was probed with appropriate antibodies and visualised using enhanced chemiluminescence (ECL) Super Signal Pico Plus (Thermo Fisher Scientific), with intensity digitally captured using a ChemiDoc Imager (Bio-Rad).

### Flow cytometry

Intensity from GFP-expressing (excitation laser 488 nm, emission filter 525/40 nm) and FM4-64-labelled (excitation laser 561 nm, emission filter 710/50 nm) live cells at room temperature was recorded using a CytoFLEX flow cytometer (Beckman Coulter), and intensity measurements from gated cells measured with FCS Express v6.0 (DeNovo software). Typically, 100,000 cells, gated for fluorescence-positive yeast cells (using forward/side scatter), were flowed at ∼600 V to maintain a rate of approximately 500–1000 cells measured per second. Unlabelled/non-expressing cells were used for background calibration. Channel recordings from other detectors (e.g. 530/50 nm) were also recorded to measure background autofluorescence. Intensity measurements are plotted. The coefficient of variation (CV) equal to the population standard deviation divided by the population mean is expressed as a percentage.

### Yeast growth and recovery assays

Yeast cultures were grown overnight in glucose-containing YPD or SC media diluted to OD_600_=0.4 and used to create 10-fold serial dilutions in a sterile 96-well plate, which were then plated on the indicated media and grown for 2 days at 30°C before growth was recorded using a Phenobooth Imager (Singer Instruments). For recovery-based assays, cells were exchanged to raffinose media for 2 h prior to equivalent cell numbers being harvested and either plated on the indicated solid media or brought up in glucose-containing minimal media containing various concentrations of uracil. OD_600_ measurements were recorded every 60 min using a MultiSkanGo plate reader and SkanIt software (Thermo Fisher Scientific).

### Statistical analyses

The statistical significance for each experimental condition (e.g. raffinose media, mutant yeast strains) in comparison to control conditions (e.g. glucose media, wild-type cells) was calculated using unpaired Holm–Sidak method *t*-tests in GraphPad Prism v8.3.1. An asterisk is used in graphs to denote *P*<0.05 or less, as indicated in figure legends, and all generated *P*-values for each experiment are included in Table S8.

## Supplementary Material

Supplementary information
