# Supplementary material for: A glucose-starvation response governs endocytic trafficking and eisosomal retention of surface cargoes in budding yeast
Source: J Cell Sci. 2021 Jan 25;134(2):jcs257733. doi: 10.1242/jcs.257733 (PMC7860119; doi:10.1242/jcs.257733)
Supplement: Supplementary information [file joces-134-257733-s1.pdf]

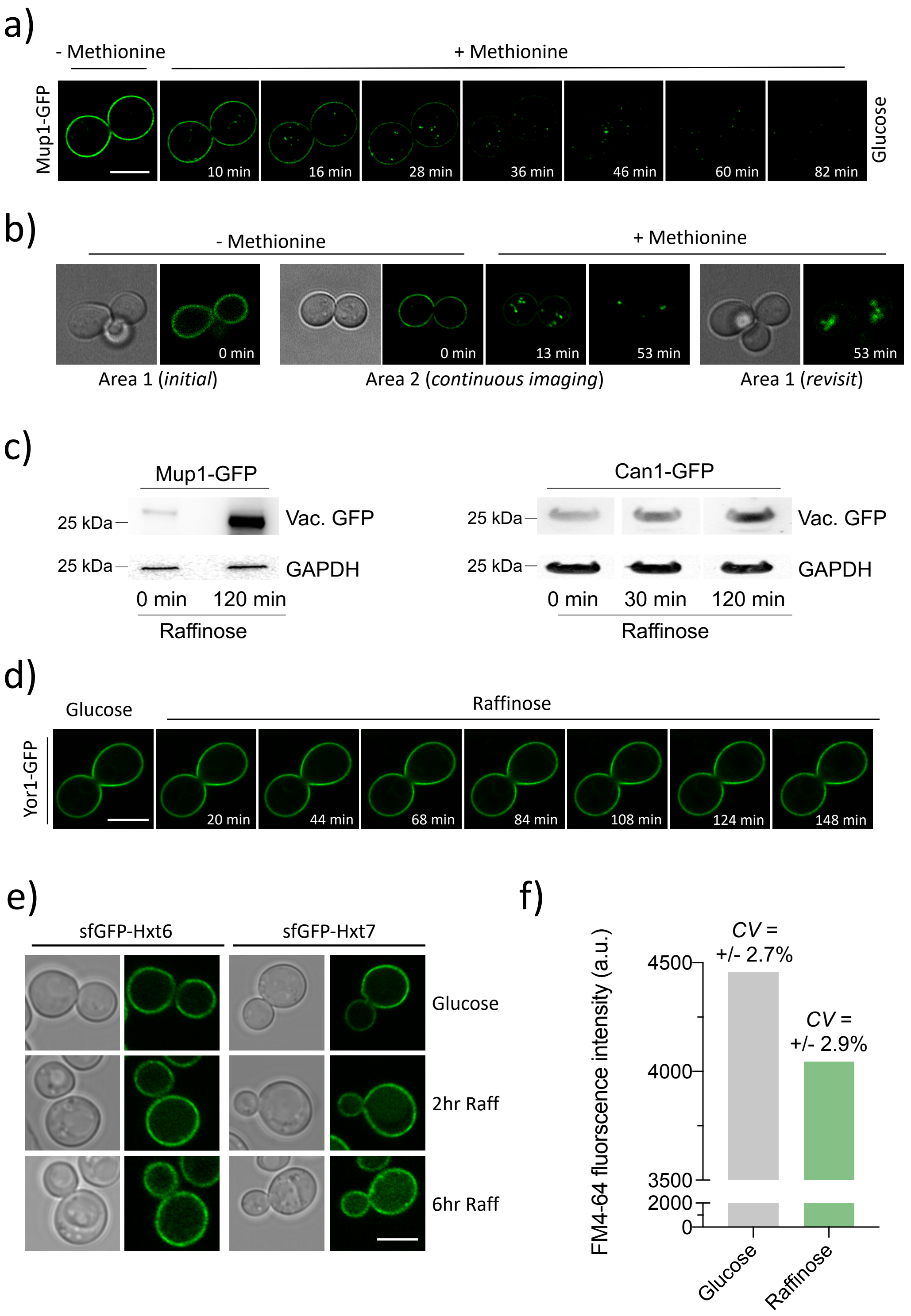

Figure S1: Differential cargo trafficking effects following glucose starvation. **a)** Wild-type cells expressing Mup1-GFP were grown to mid-log phase in SC media lacking methionine, processed for time-lapse microscopy and then imaged every 2 minutes following addition of 20 µg/ml methionine. **b)** Vacuolar GFP bleaching in wild-type cells expressing Mup1-GFP grown to mid-log phase in SC media lacking methionine and processed for time-lapse microscopy. Area 1 was imaged before addition of methionine, before moving to a distinct region of the same plate (Area 2) for continuous imagining from 0 – 53 minutes of methionine addition. Following this period, Area1 was re-visited and imaged to show the difference in photobleaching of vacuolar sorted Mup1-GFP. **c)** Levels of vacuolar processed GFP from cargo (Mup1-GFP, left and Can1-GFP, right) expressing cells were assessed in glucose and raffinose treated cells by immunoblotting lysates with GFP antibodies. Loading was assessed with anti-GAPDH antibodies. **d)** Wild-type cells expressing Yor1-GFP from the *CUP1* promoter by addition of 50µM copper chloride were grown to mid-log phase in glucose containing media, processed for time-lapse microscope and imaged for indicated time course after exchange with raffinose media. **e)** Strains expressing GFP tagged Hxt6 and Hxt7 expressed from the *NOP1* promoter were grown in glucose or raffinose media for indicated periods prior to fluorescence microscopy. **f)** Wild-type cells grown either in glucose media or exchanged with raffinose for 15 minutes were incubated with YPD containing 40 µM FM4-64 dye for 4 minutes at room temperature before ice cold washes were performed with minimal media to remove excess dye. Mean fluorescence of ~10,000 cells was then measured by flow cytometry, plotted with coeffecint of variation (*cv*) indicated. Scale bar, 5 µM.

Journal of Cell Science • Supplementary information

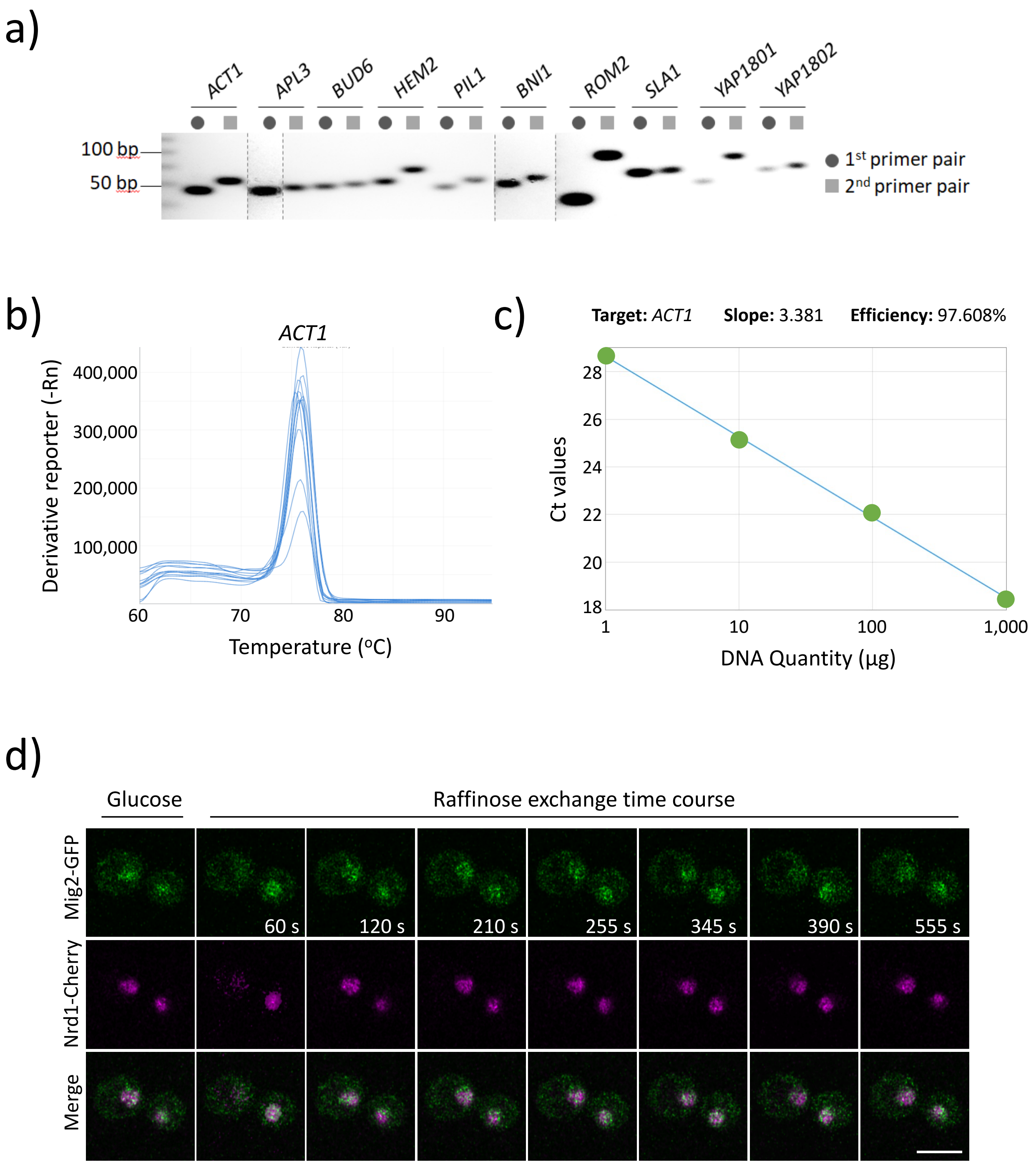

Figure S2: qPCR optimisation and time-lapse microscopy of Mig2-GFP: **a)** Indicated oligo pairs were used to generate PCR products from a gDNA template and analysed by agarose gel of primer pair validation of the qPCR using wild-type cells mRNA. **b)** Melt curve analyses for all qPCR primer pairs were carried out to ensure no primer dimer species were detected, read out shows example for ACT1. **c)** Primer pair efficiency was also performed for all primer pairs used in this study (shown for ACT1), with ΔCt values across a 10-fold serial dilution plotted (slope = -3.33 equivalent to 100% efficiency). Data for all primer pair analysis is recorded in Supplemental Tables S2 & S3. **d)** Wild-type cells expressing Mig2-GFP and Nrd1-mCherry were grown to mid log phase in minimal media prior to processed for time-lapse microscopy. Images were captured every 5 seconds following raffinose exchange, with representative time-slices shown. Scale bar, 5 µM.

Journal of Cell Science • Supplementary information

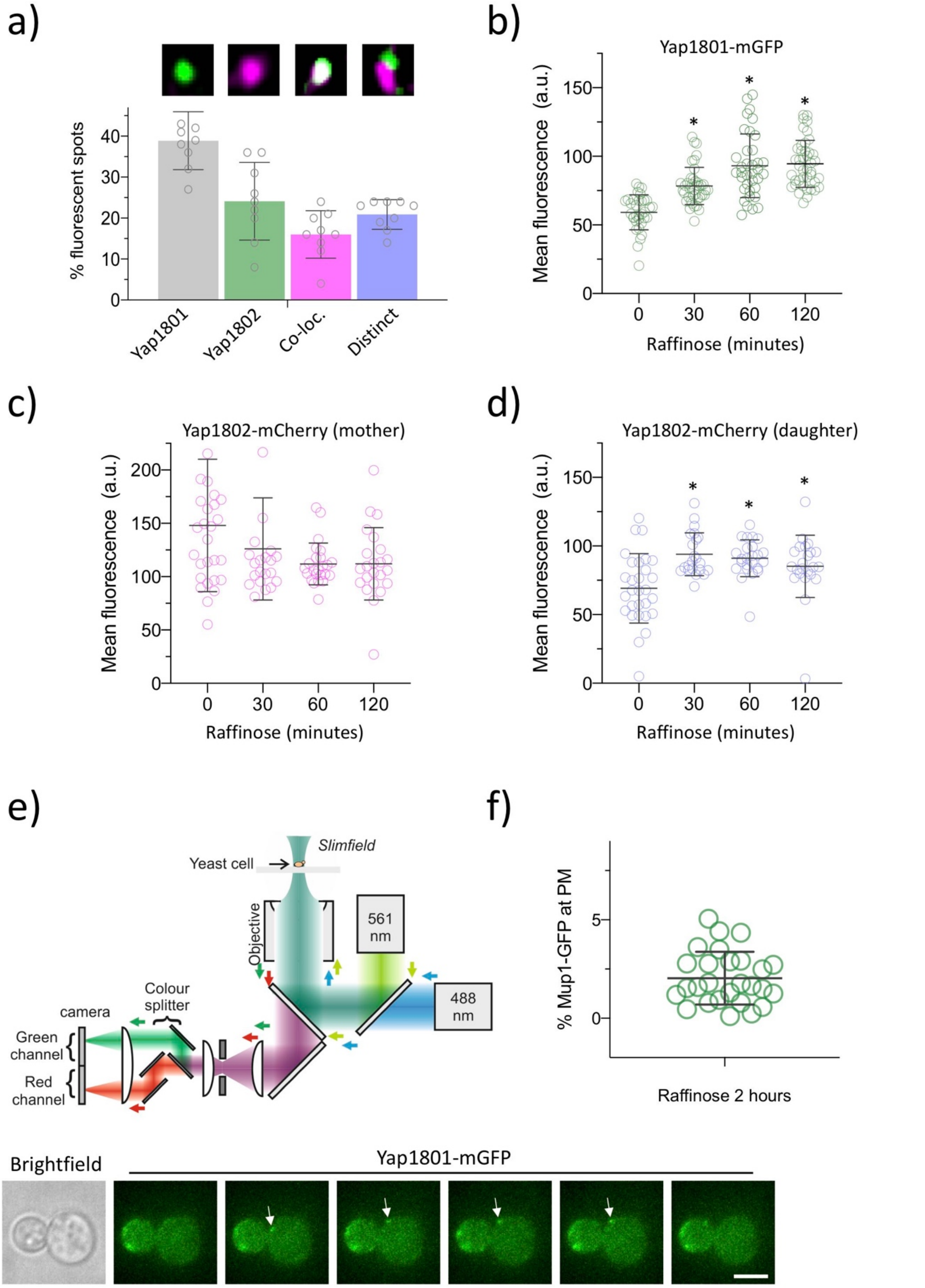

Figure S3: Localisation of Yap1801 and Yap1802: **a)** Histogram showing the co-localisation analysis of Airyscan confocal images of Yap1801-mGFP and Yap1802-mCherry expressing wild-type cells grown to mid-log phase in SC selective media. Error bars showing standard deviation (n = 235 foci analysed). Examples of each localisation category is shown (upper). **b-d)** Histograms showing mean fluorscence from confocal images cells grown in indicated media condtions whlst expressing **b)** Yap1801-mGFP (total cell), **c)** Yap1802-mCherry (just mother cell), **d)** Yap1802-mCherry (just daughter cell). Intensity was averaged from n=>36 cells per condition over 3 biological replicates, with error bars showing standard deviation. **e)** Slimfield microscopy, schematic diagram showing set-up for dual-colour imaging of yeast cells. Lower panels show Yap1801-mGFP fluorescent spot (white arrow) tracking from images acquired every 5ms. **f)** Percentage plasma membrane localised Mup1-GFP was caculated from cells grown in raffinose for two hours. * indicates Student *t*-test p-values <0.05. Scale bar, 5 μM.

Journal of Cell Science • Supplementary information

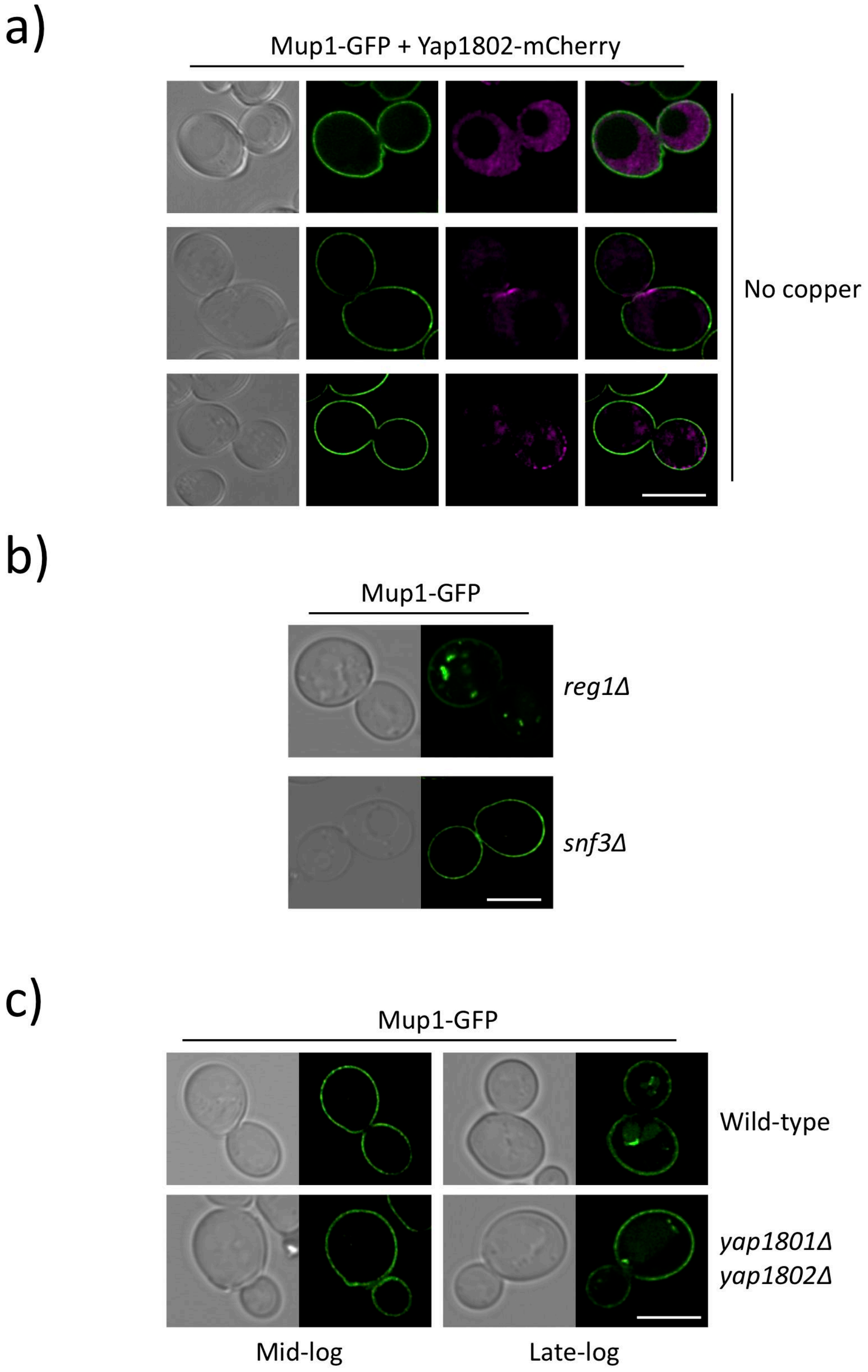

Figure S4: Functional relationship between Mup1-GFP trafficking and yeast AP180s: a) Representative examples of different Yap1802-mCherry localisations are shown from expression under the *CUP1* promoter with no additional copper added to the media, in cells co-expressing Mup1-GFP. b) Indicated mutant null strains expression Mup1-GFP were imaged by confocal microscopy. c) Mup1-GFP localisation was imaged in wild-type and *yap1801Δ yap1802Δ* cells grown to mid-log (OD_{600} = 1.0) and late-log (OD_{600} = 2.0) phase.

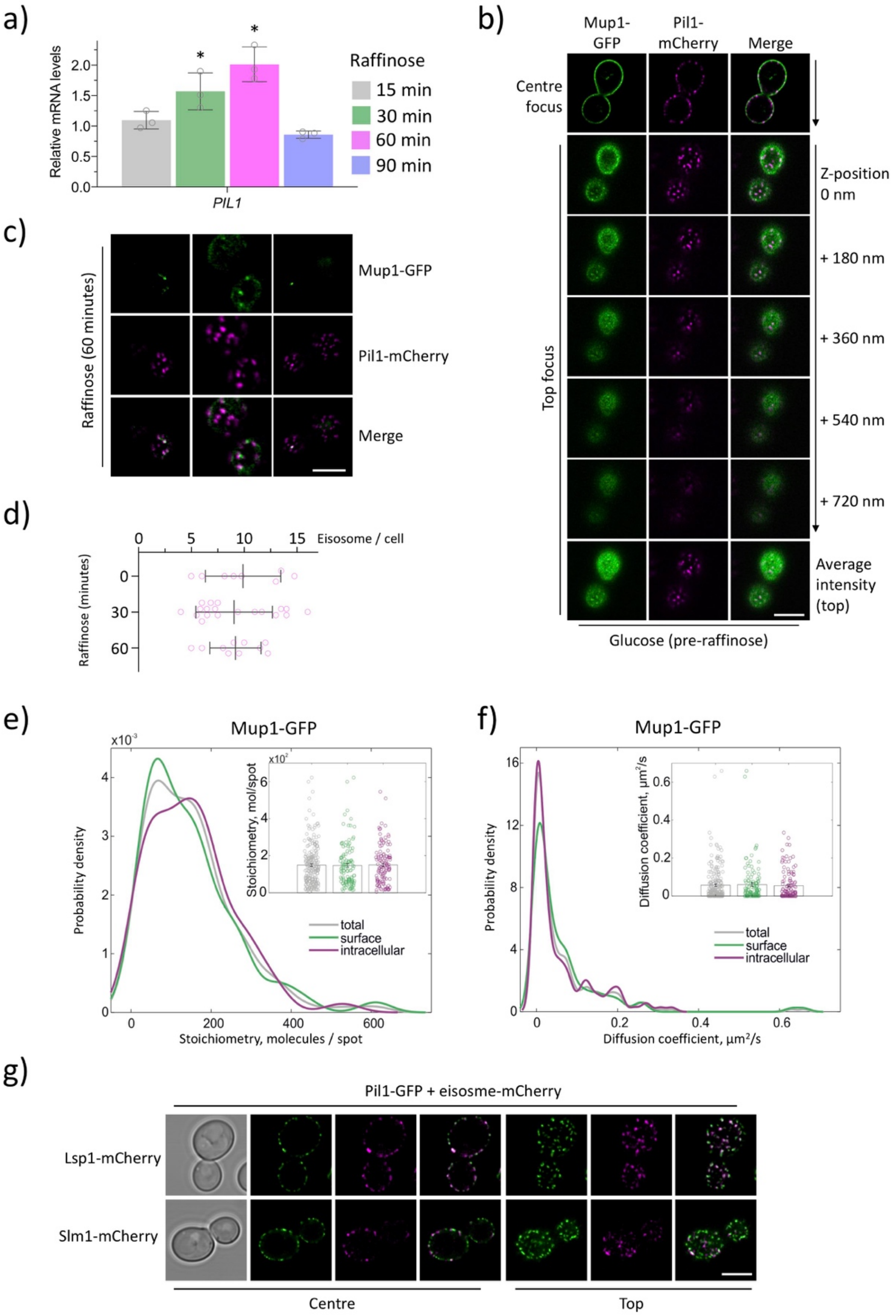

Figure S5: Analyses of eisosomes in response to changes in glucose levels: **a)** Quantitative RT-PCR of *PIL1* performed from RNA extracted from wild-type cells grown in glucose media and relative levels compared to cells grown in raffinose media for indicated time course. Error bars show the standard deviation from 3 biological replicates (each averaged from 3 technical replicates). **b)** Wild-type cells expressing Pil1-mCherry and Mup1-GFP were grown to mid-log phase in SC media and prepared for confocal imaging. Cells were focussed to the top of the cell and imaged at 0.18µm intervals in the z-axis. An average intensity projection from the indicated 5 slices is shown (lower panel). **c)** Top focus 3D confocal imaging of cells expressing Mup1-GFP and Pil1-mCherry after 60 minutes raffinose starvation. **d)** Number of Pil1-mCherry marked eisosomes per cell in glucose, 30-minutes or 60-minutes raffinose and depicted with standard deviation error bars. **e)** Kernel density plots of Mup1-GFP stoichiometry distribution of fluorescent foci in the whole cell (grey), on the surface (green) or inside the cell (purple). Inserts: jitter plots of stoichiometries of fluorescent foci detected in the whole cell, on the surface or inside the cell. Error bars represent standard error. **f)** Kernel density plots of Mup1-GFP diffusion coefficients, using same labelling as (e) for total, surface and intracellular signal. **g)** Cells co-expressing Pil1-GFP with either Lsp1-mCherry (upper) or Slm1-mCherry (lower) were imaged at centre and top focus by confocal microscopy. * indicates Student *t*-test p-values <0.05. Scale bar, 5 µm

Journal of Cell Science • Supplementary information

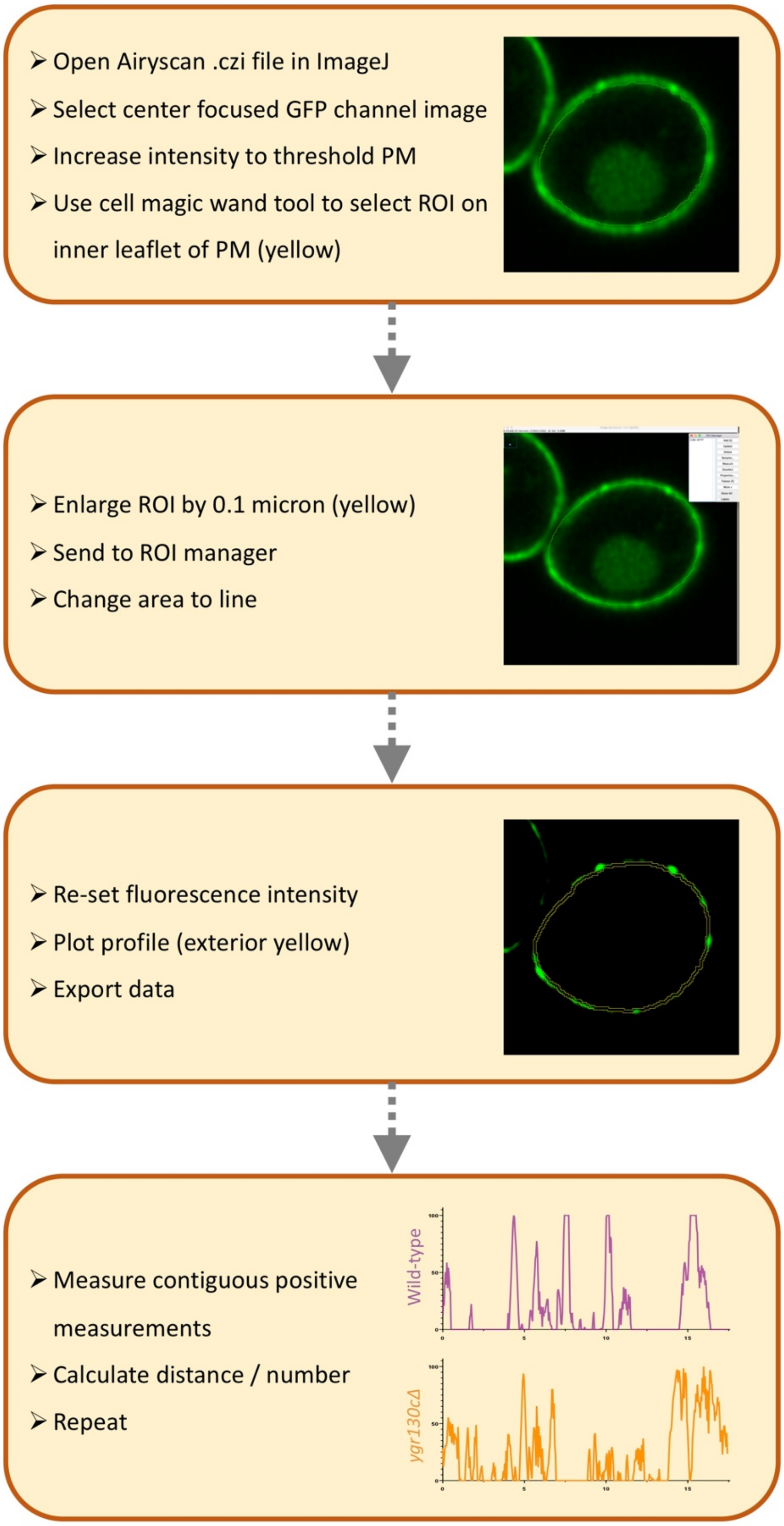

Figure S6: Analyses of eisosomes in response to changes in glucose levels: Workflow for systematic image processing used to measure the length of contiguous GFP signal at the plasma membrane in wild-type and *ygr130c*Δ yeast cells expressing Mup1-GFP.

Journal of Cell Science • Supplementary information

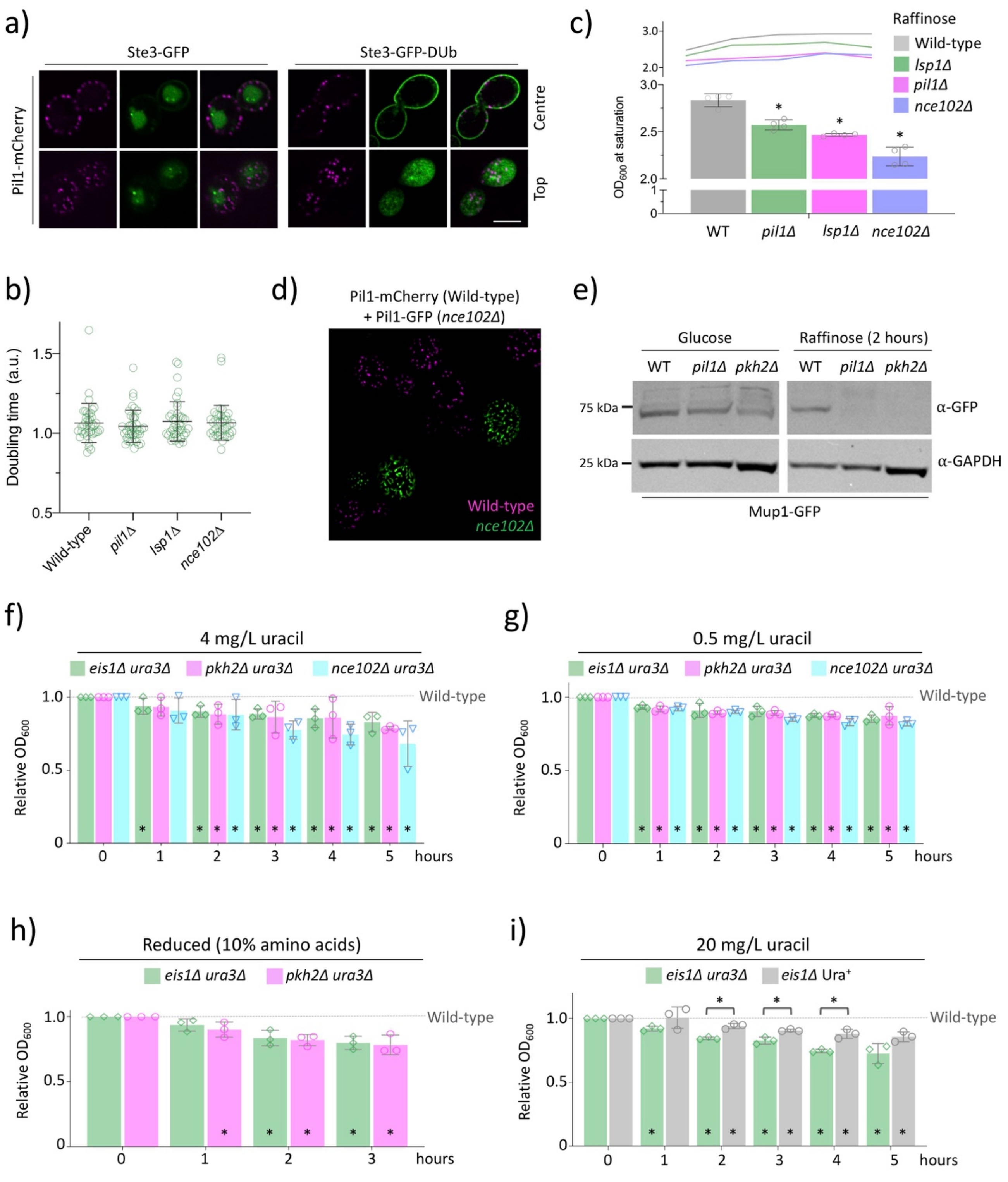

Figure S7: The role of eisosomes in cargo specific retention following starvation. **a)** Wild-type cells co-expressing Pil1-mCherry and either Ste3-GFP (left) or Ste3-GFP-DUb (right) were imaged by 3D confocal Airyscan microscopy. **b)** Indicated strains were grown to mid-log phase overnight and allowed to reach saturation, with OD_{600} measurements captured every hour, depicted as a line graph (upper), or the maximum OD_{600} shown as a histogram (lower). **c)** Triplicate cultures were grown to mid-log phase overnight, diluted to low optical density and then grown in 5ml cultures. Samples were taken for OD_{600} measurements every hour over 8 – 10 hours and used to calculate average doubling times, with distribution displayed in scatter plot. **d)** Wild-type cells expressing Pil1-mCherry were co-cultured with *nce102Δ* cells expressing Pil1-GFP before Airyscan microscopy and merging colour channels. **e)** Indicated strains expressing Mup1-GFP were grown in either glucose (left) or 2 hours raffinose (right) media before denatured lysates generated and analysed by SDS-PAGE and immunoblot with antibodies against GFP and GAPDH. **f -i)** Indicated strains were grown to mid-log phase before incubation in raffinose media for two hours. Cells were then resuspended in SC media containing glucose and 4 mg/L Uracil **(f)**, 0.5 mg/L Uracil, **(h)** SC-media supplemented with only 10% amino acids or **(i)** 20 mg/L Uracil before OD_{600} measurements recorded at 1-hour intervals during recovery period, depicted as histogram. Error bars show standard deviation from 3 biological replicates. * indicates Student *t*-test p-values <0.05. Scale bare, 5 µm.

Table S1: **Functional clustering of Mig1 candidates**

Click here to Download Table S1

Table S2: **Ygr130c interactome**

Click here to Download Table S2

Table S3: **Mup1 interactome**

Click here to Download Table S3

Table S4: **Yeast Strains used in this study**

Click here to Download Table S4

Table S5: **Plasmids used in this study**

Click here to Download Table S5

Table S6: **qPCR primers**

Click here to Download Table S6

Table S7: **Primer efficiency and usage**

Click here to Download Table S7

Table S8: **Statistical analyse**

Click here to Download Table S8

Journal of Cell Science • Supplementary information

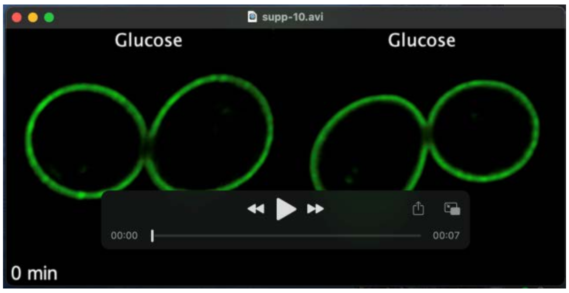

Movie 1: **Mup1-GFP endocytosis is triggered in raffinose, but not glucose, media**

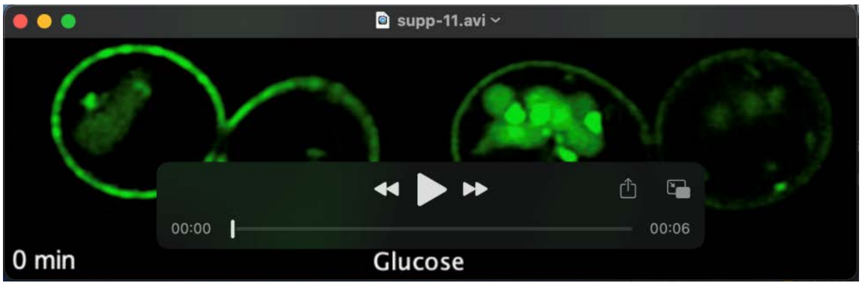

Movie 2: **Endocytic trafficking of Fur4-mNG / Ste3-GFP following raffinose exchange**

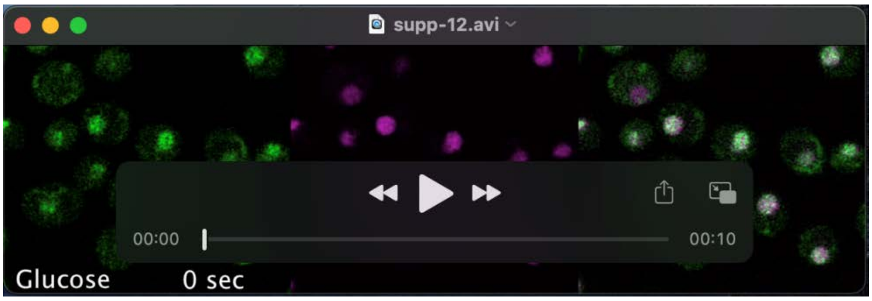

Movie 3: **Raffinose exchange triggers Mig1-mGFP translocation from Nrd1-mCherry labelled nuclei**

Journal of Cell Science • Supplementary information

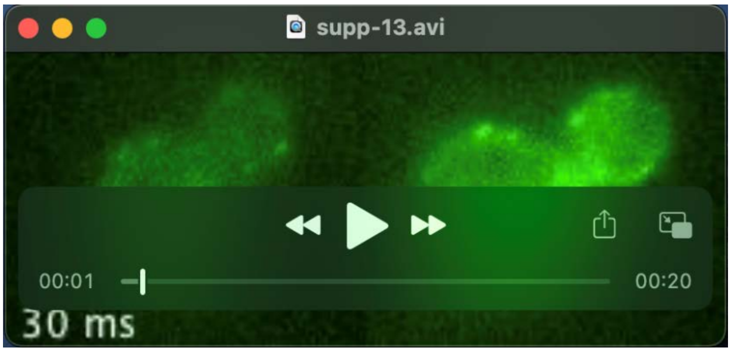

Movie 4: **Slimfield acquisition of cells expressing mGFP tagged versions of Yap1801 and Yap1802**

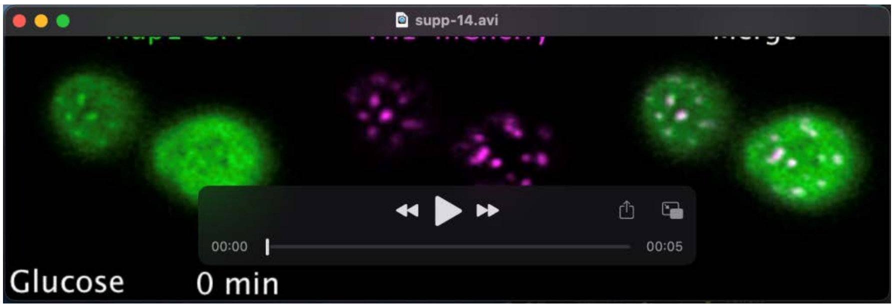

Movie 5: **Mup1-GFP accumulates in Pil1-mCherry eisosomes following raffinose exchange**

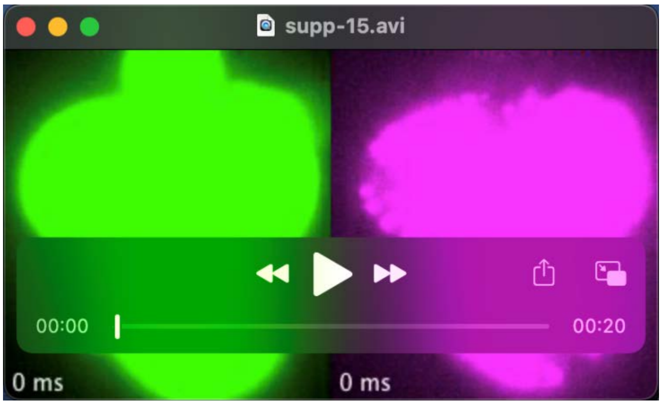

Movie 6: **Slimfield acquisition of cells co-expressing Mup1-GFP and Pil1-mCherry**

SUPPLEMENTAL REFERENCES

Adell, M. A. Y., Migliano, S. M., Upadhyayula, S., Bykov, Y. S., Sprenger, S., Pakdel, M., Vogel, G. F., Jih, G., Skillern, W., Behrouzi, R., et al. (2017). Recruitment dynamics of ESCRT-III and Vps4 to endosomes and implications for reverse membrane budding. *Elife* **6**, e31652.

Brachmann, C. B., Davies, A., Cost, G. J., Caputo, E., Li, J., Hieter, P. and Boeke, J. D. (1998). Designer deletion strains derived from Saccharomyces cerevisiae S288C: a useful set of strains and plasmids for PCR-mediated gene disruption and other applications. *Yeast* **14**, 115 132.

MacDonald, C., Payne, J. A., Aboian, M., Smith, W., Katzmann, D. J. and Piper, R. C. (2015). A Family of Tetraspans Organizes Cargo for Sorting into Multivesicular Bodies. *Dev Cell* **33**, 328 342.

Robinson, J. S., Klionsky, D. J., Banta, L. M. and Emr, S. D. (1988). Protein sorting in Saccharomyces cerevisiae: isolation of mutants defective in the delivery and processing of multiple vacuolar hydrolases. *Molecular and Cellular Biology* **8**, 4936 4948.

Sikorski, R. S. and Hieter, P. (1989). A system of shuttle vectors and yeast host strains designed for efficient manipulation of DNA in Saccharomyces cerevisiae. *Genetics* **122,** 19-27.

Stringer, D. K. and Piper, R. C. (2011). A single ubiquitin is sufficient for cargo protein entry into MVBs in the absence of ESCRT ubiquitination. *J Cell Biology* **192**, 229 242.

Urbanowski, J. L. and Piper, R. C. (2001). Ubiquitin Sorts Proteins into the Intralumenal Degradative Compartment of the Late-Endosome/Vacuole. *Traffic* **2**, 622 630.

Weill, U., Yofe, I., Sass, E., Stynen, B., Davidi, D., Natarajan, J., Ben-Menachem, R., Avihou, Z., Goldman, O., Harpaz, N., et al. (2018). Genome-wide SWAp-Tag yeast libraries for proteome exploration. *Nat Methods* **15**, 617–622.

Winzeler, E. A., Shoemaker, D. D., Astromoff, A., Liang, H., Anderson, K., Andre, B., Bangham, R., Benito, R., Boeke, J. D., Bussey, H., et al. (1999). Functional characterization of the S. cerevisiae genome by gene deletion and parallel analysis. *Science* **285**, 901 906.

Wollman, A. J., Shashkova, S., Hedlund, E. G., Friemann, R., Hohmann, S. and Leake, M. C. (2017). Transcription factor clusters regulate genes in eukaryotic cells. *Elife* **6**, e27451.

Xu, P., Hankins, H. M., MacDonald, C., Erlinger, S. J., Frazier, M. N., Diab, N. S., Piper, R. C., Jackson, L. P., MacGurn, J. A. and Graham, T. R. (2017). COPI mediates recycling of an exocytic SNARE by recognition of a ubiquitin sorting signal. *Elife* **6**, e28342.

Journal of Cell Science • Supplementary information
